# Identification of Natural Products Inhibiting SARS-CoV-2 by Targeting Viral Proteases: A Combined in Silico and in Vitro Approach

**DOI:** 10.1021/acs.jnatprod.2c00843

**Published:** 2023-01-18

**Authors:** Andreas Wasilewicz, Benjamin Kirchweger, Denisa Bojkova, Marie Jose Abi Saad, Julia Langeder, Matthias Bütikofer, Sigrid Adelsberger, Ulrike Grienke, Jindrich Cinatl Jr., Olivier Petermann, Leonardo Scapozza, Julien Orts, Johannes Kirchmair, Holger F. Rabenau, Judith M. Rollinger

**Affiliations:** † Department of Pharmaceutical Sciences, University of Vienna, Josef-Holaubek-Platz 2, 1090 Vienna, Austria; ‡ Vienna Doctoral School of Pharmaceutical, Nutritional and Sport Sciences, University of Vienna, Josef-Holaubek-Platz 2, 1090 Vienna, Austria; § Institute of Medical Virology, University Hospital Frankfurt, Paul-Ehrlich-Straße 40, 60596 Frankfurt am Main, Germany; ⊥ Swiss Federal Institute of Technology, Laboratory of Physical Chemistry, ETH Zurich, Vladimir-Prelog-Weg 1-5/10, 8093 Zurich, Switzerland; ∥ Pharmaceutical Biochemistry Group, School of Pharmaceutical Sciences, University of Geneva, 1205 Geneva, Switzerland; ○ Institute of Pharmaceutical Sciences of Western Switzerland, University of Geneva, 1205 Geneva, Switzerland

## Abstract

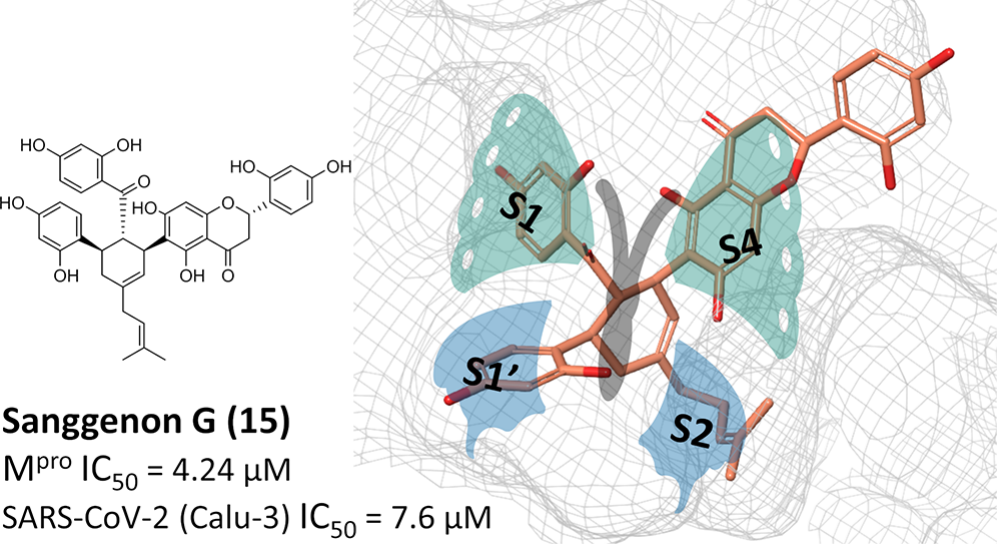

In this study, an integrated in silico–in vitro approach was employed to discover natural products (NPs) active against SARS-CoV-2. The two SARS-CoV-2 viral proteases, i.e., main protease (M^pro^) and papain-like protease (PL^pro^), were selected as targets for the in silico study. Virtual hits were obtained by docking more than 140,000 NPs and NP derivatives available in-house and from commercial sources, and 38 virtual hits were experimentally validated in vitro using two enzyme-based assays. Five inhibited the enzyme activity of SARS-CoV-2 M^pro^ by more than 60% at a concentration of 20 μM, and four of them with high potency (IC_50_ < 10 μM). These hit compounds were further evaluated for their antiviral activity against SARS-CoV-2 in Calu-3 cells. The results from the cell-based assay revealed three mulberry Diels–Alder-type adducts (MDAAs) from *Morus alba* with pronounced anti-SARS-CoV-2 activities. Sanggenons C (**12**), O (**13**), and G (**15**) showed IC_50_ values of 4.6, 8.0, and 7.6 μM and selectivity index values of 5.1, 3.1 and 6.5, respectively. The docking poses of MDAAs in SARS-CoV-2 M^pro^ proposed a butterfly-shaped binding conformation, which was supported by the results of saturation transfer difference NMR experiments and competitive ^1^H relaxation dispersion NMR spectroscopy.

Since the outbreak of the COVID-19 pandemic, more than 6.4 million people worldwide have died by being infected with severe acute respiratory syndrome coronavirus 2 (SARS-CoV-2).^1^ The rapid development and availability of safe and effective vaccines represented a giant leap toward global immunity and significantly reduced the severity of the pandemic. However, disruptive seasonal waves of COVID-19 outbreaks are expected to contribute to a significant death toll in the future, comparable to seasonal influenza epidemics, which claim roughly 145,000 lives per year.^2^ In addition, it is likely that new variants of SARS-CoV-2 will emerge that escape the immune system or increase transmissibility.^3^ Hence, an additional cornerstone to combat COVID-19 besides vaccination and hygiene measures are antiviral drugs for the treatment of acute infections.^4,5^ Within the last years, several small-molecule drugs and monoclonal antibodies have become available for the treatment of SARS-CoV-2. The nucleoside analogues remdesivir (**1**) and molnupiravir (**2**) are two repurposed drugs that have been developed initially for the treatment of Ebola virus and Venezuelan equine encephalitis virus, respectively (Chart 1).^6^ The most recently approved drug, nirmatrelvir (**3**), targets the viral main protease (M^pro^; 3C-like protease, nsp5) and was developed using a structure-based design approach.^7^ Its clinical efficacy substantiates SARS-CoV-2 proteases as promising targets for drug discovery, similar to other viral infections that can be treated by targeting viral proteases.^8^ For the present in silico study, the two SARS-CoV-2 proteases, M^pro^ and papain-like protease (PL^pro^; domain of nsp3), were selected as targets. Both are cysteine proteases and essential for virus replication, as they cleave the viral polyprotein on distinct sites.^9^ While M^pro^ leads to the formation of nonstructural proteins (nsp) 4–16,^10^ PL^pro^ splits the polyprotein into nsp 1–3.^11^ In addition, PL^pro^ also possesses deubiquitination and deISGylation activities that cause the release of K48-Ub2 and ISG15 from host cell proteins. Since both K48-Ub2 and ISG15 result in important cell signals upon infection, PL^pro^ is further able to counteract the host cell immune response.^12^

**Chart 1:**
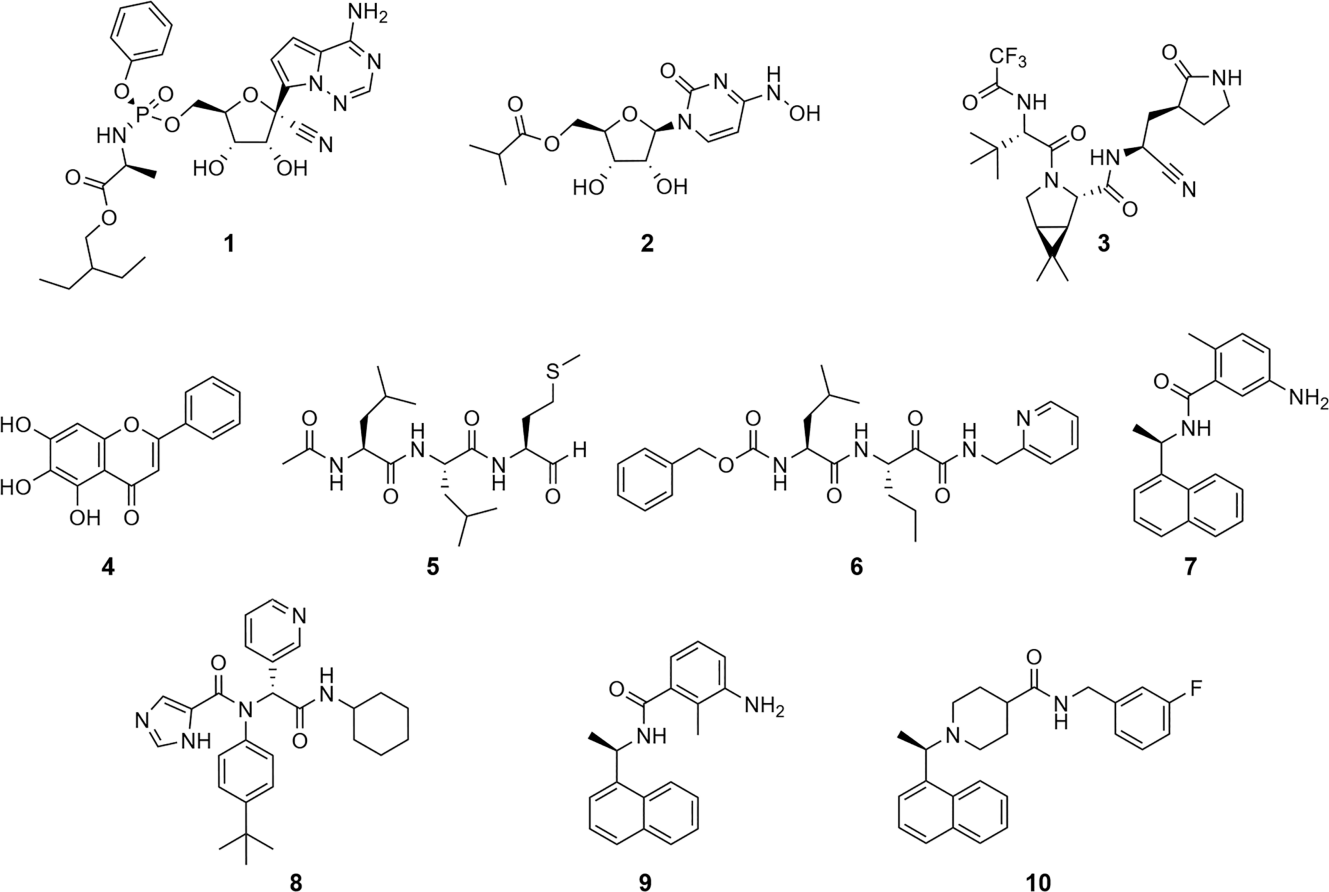
Chemical Structures of Approved Drugs against SARS-CoV-2 (**1**–**3**), Reported Inhibitors of M^pro^ and PL^pro^ Used as Positive Controls (**4–7**), and Co-Crystallized Ligands of the Protein Structures Used as Queries (**8**–**10**)

For both proteases, several structures including a bound inhibitor derived by X-ray crystallography are available from the Protein Data Bank (PDB). This allowed for the implementation of a structure-based in silico workflow for the identification of promising inhibitors of the SARS-CoV-2 proteases by virtual screening of natural product (NP) databases.

The focus on NPs was set as they are the most prolific source of new anti-infective drugs.^13^ Some NPs were already reported to inhibit SARS-CoV-2 in vitro, e.g., oridonin,^14^ cannabinoid acids,^15^ and gallinamide A.^16^ Baicalein (**4**) and andrographolide are also described as M^pro^ inhibitors with anti-SARS-CoV-2 activity.^17,18^

To discover NPs active against SARS-CoV-2, selected virtual hits (VHs) were experimentally validated for their inhibitory activity in enzyme-based assays and further tested for their antiviral effect in Calu-3 cells. Herein, mulberry Diels–Alder-type adducts (MDAAs) were discovered as a new class of M^pro^ inhibitors with potent anti-SARS-CoV-2 activities. Saturation transfer difference (STD) NMR experiments were conducted to corroborate the docking poses predicted in silico. STD amplification factors (STD-AF) gave insights into ligand–target interactions.

## Results and Discussion

### Molecular Docking

**Chart 2:**
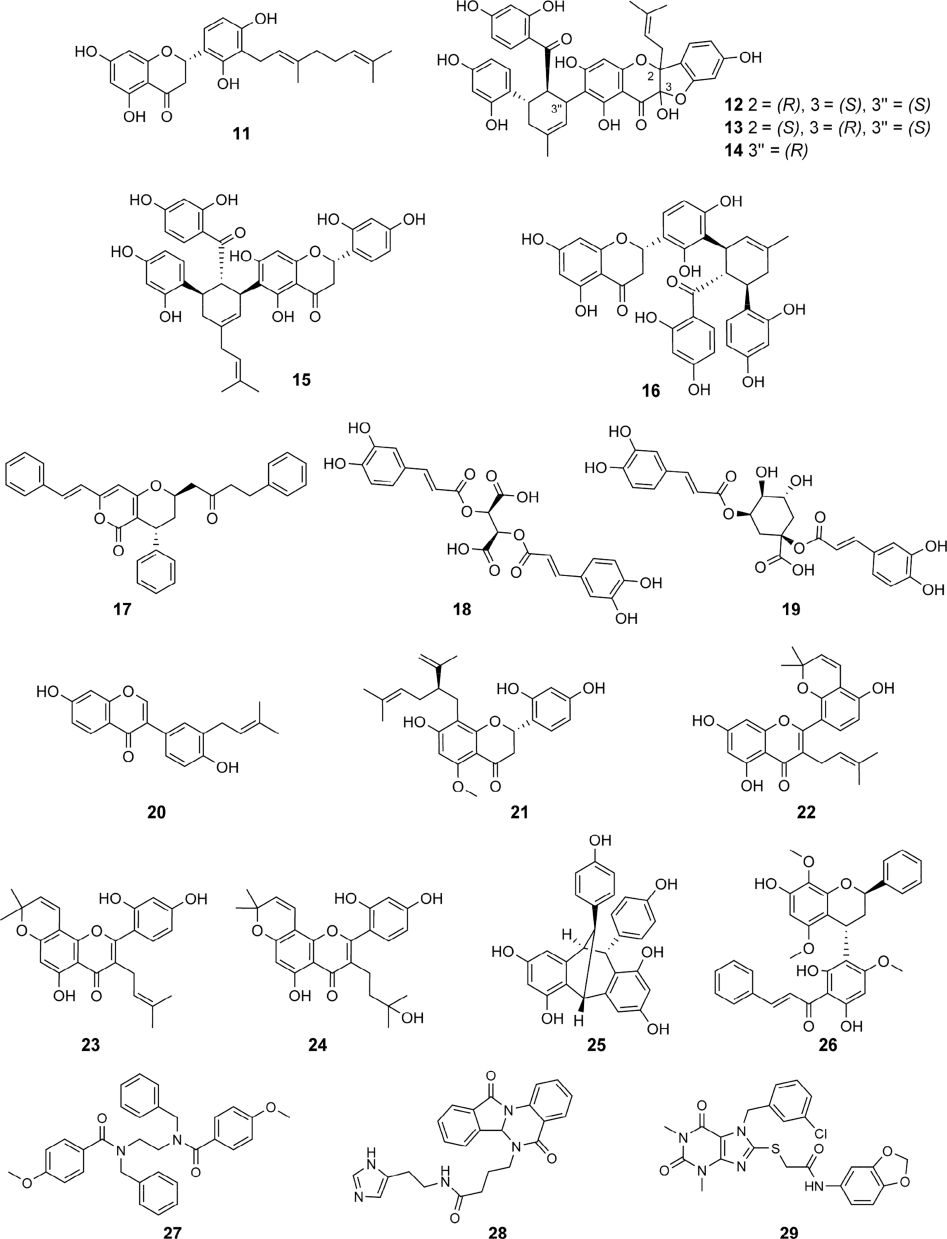


**Chart 3:**
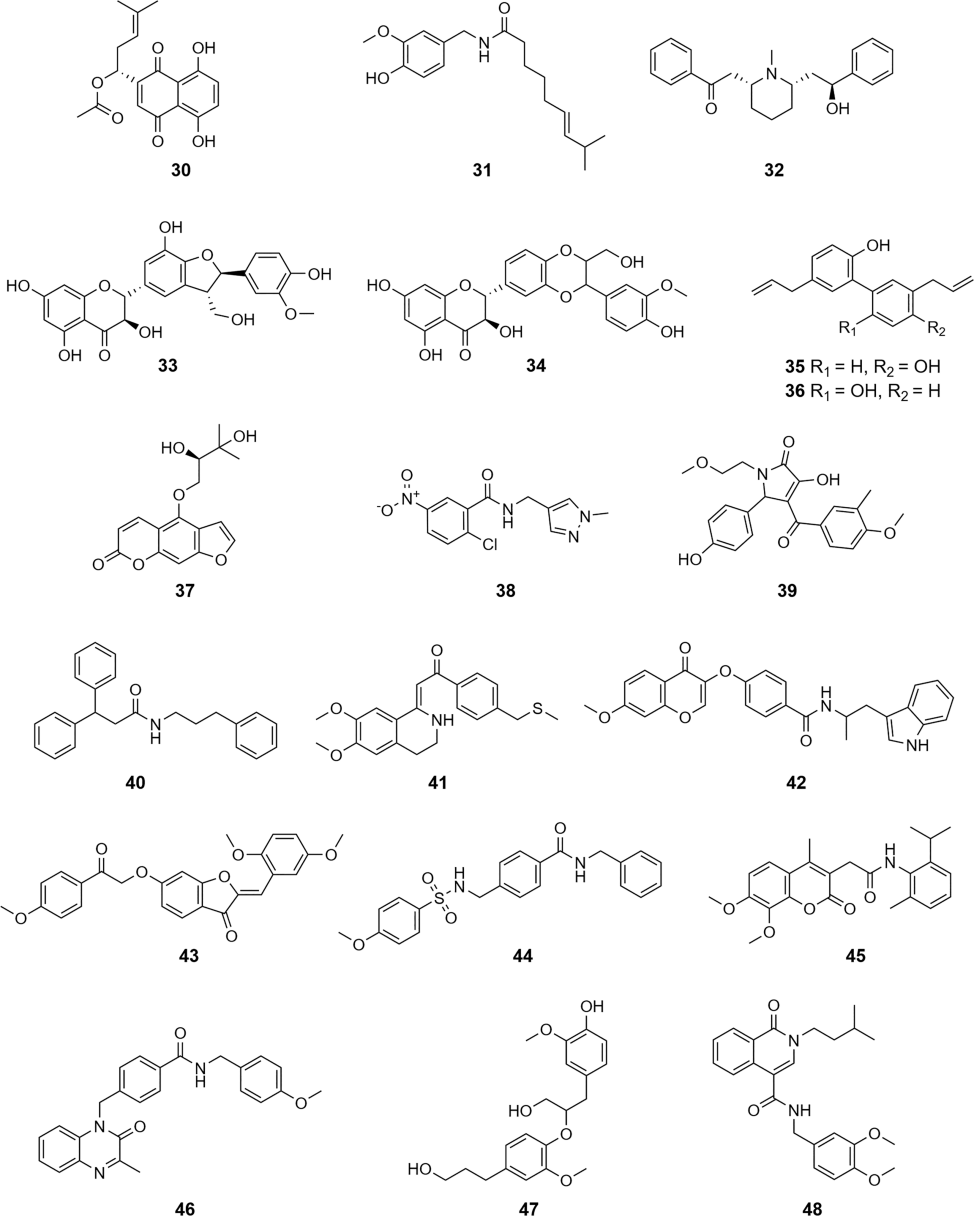


A structure-based in silico approach was employed to identify NPs and NP derivatives with a high probability to competitively inhibit the SARS-CoV-2 protease M^pro^ or PL^pro^. Three protein structures were selected for virtual screening: one structure for M^pro^ (PDB code: 6W63) and two structures for PL^pro^ (PDB codes: 7JN2 and 4OW0).^19−21^ For PL^pro^, two different structures were selected to consider the conformational flexibility of Tyr268 and Gln269, which are in proximity to the binding site (Figure S1, Supporting Information). Other major conformational changes were not observed in any of the X-ray structures of M^pro^ and PL^pro^ available at that time (October 2020). The fact that 4OW0 is an X-ray structure of SARS-CoV PL^pro^ and not SARS-CoV-2 PL^pro^ was regarded as negligible given their structurally similar binding site.^22^ Docking of two NP databases against the substrate sites of the three enzyme structures was carried out with GOLD.^23^ The databases used were (i) an in-house database (IHDB) of 1309 NPs that are physically available at the Department of Pharmaceutical Sciences, Division of Pharmacognosy, University of Vienna,^24^ and (ii) the Molport Natural Products Database (MNPDB), comprising 140,164 commercially available NPs and NP derivatives.^25^ Docking poses were ranked with the ChemPLP scoring function. Among the top-ranked docking poses, altogether 61 compounds were preselected by considering (i) the predicted protein–ligand interactions, (ii) the predicted binding site occupation, and (iii) the pose reproduction and comparison with that of known inhibitors (X77 (**8**), Y41 (**9**), S88 (**10**)) (Table S1, Supporting Information).^26^ For the final selection of VHs to be purchased and experimentally validated, the following criteria were employed: (i) availability of high-quality compounds (purity ≥95%, according to the vendor), (ii) low probability of assay interference as assessed by Hitdexter 2.0^27^ and FAFDrugs 4,^28^ and (iii) relevance as known constituents of herbal remedies that are used traditionally for the treatment of respiratory infections.^29^ Tables reporting all VHs including SMILES notations and information on potential assay interferences are provided in Tables S2–S4 (Supporting Information). Finally, 19 VHs were selected for testing in an M^pro^ enzyme inhibition assay (Chart 2), and a further 19 VHs were selected for evaluation in a PL^pro^ enzyme inhibition assay (Chart 3).

### Experimental Validation

For validation of the virtual predictions, the 19 M^pro^ VHs, **11**–**29**, were tested for their potential to inhibit the protease activity of SARS-CoV-2 M^pro^ at 20 and 100 μM using a fluorescence resonance energy transfer quenching assay with 5-FAM-AVLQSGFR-Lys(Dabcyl)-K-amide as substrate. For validation of PL^pro^ inhibition, compounds **30**–**48** were tested in a fluorogenic peptide cleavage assay at 20 and 100 μM using Z-RLRGG-AMC as substrate. In Tables 1 and 2, the inhibitory activities of the respective VHs against SARS-CoV-2 M^pro^ and PL^pro^ are reported. Considering an efficacy threshold of 60% enzyme inhibition at 20 μM, a hit rate of 13% was achieved.

**Table 1:** Evaluation of Inhibitory Activities of VHs for SARS-CoV-2 M^pro^

			SARS-CoV-2 M^pro^		
			enzyme inhibition (%)		
compound no.	compound name		100 μM	20 μM	IC_50_ (μM)
**4**	baicalein	pos. control	90.0 ± 1.4	8.6^a^	50.6
**5**	calpain inhibitor II	pos. control			2.0
**6**	calpain inhibitor XII	pos. control			1.0
**11**	sanggenol A	VH	98.3 ± 2.4	31.9^a^	
**12**	sanggenon C	VH	99.5 ± 0.07	63.0^a^	7.2
**13**	sanggenon O	VH	99.4 ± 0.9	76.2^a^	6.7
**14**	sanggenon D	VH	98.3 ± 2.2	76.5^a^	13.9
**15**	sanggenon G	VH	99.6 ± 0.2	92.6^a^	4.8
**16**	kuwanon L	VH	97.6 ± 2.5	70.4^a^	5.3
**17**	katsumadain A	VH	47.7 ± 2.8	5.0^a^	
**18**	chicoric acid	VH	11.5 ± 0.6	0.7^a^	
**19**	cynarine	VH	9.8 ± 13.3	ND	
**20**	neobavaisoflavone	VH	84.9 ± 6.3	18.0^a^	
**21**	kurarinone	VH	99.2 ± 2.5	30.2^a^	
**22**	kuwanon A	VH	79.2 ± 2.4	45.5 ± 7.1	35.2
**23**	morusin	VH	84.0 ± 0.4	42.0 ± 9.6	33.5
**24**	morusinol	VH	71.2 ± 1.9	23.1 ± 2.8	
**25**	ampelopsin F	VH	37.5 ± 0.8	18.9 ± 2.2	
**26**	sarcandrone B	VH	7.5 ± 2.5	1.2 ± 2.5	
**27**	Molport-000-689-498	VH	28.2 ± 8.3	4.8 ± 4.0	
**28**	Molport-035-701-904	VH	59.7 ± 1.0	14.1 ± 5.1	
**29**	Molport-002-206-761	VH	7.3 ± 10.2	ND	

a For these values, the relative standard deviation of the fluorescence resonance energy transfer quenching assay of 6% applies; ND, not determinable.

**Table 2:** Evaluation of Inhibitory Activities of VHs for SARS-CoV-2 PL^pro^

			SARS-CoV-2 PL^pro^		
			enzyme inhibition (%)		
compound no.	compound name		100 μM	20 μM	IC_50_ (μM)
**7**	GRL-0617	pos. control		95.8 ± 1.0	0.8
**30**	acetylshikonin	VH	84.3 ± 4.6	41.7 ± 3.8	24.3
**31**	*trans*-capsaicin	VH	5.3 ± 0.1	5.6 ± 9.7	
**32**	lobelin	VH	14.6 ± 7.7	3.7 ± 2.6	
**33**	silicristin	VH	19.8 ± 2.0	3.4 ± 7.8	
**34**	silibinin	VH	16.8 ± 3.0	6.5 ± 3.6	
**35**	honokiol	VH	5.3 ± 3.2	4.9 ± 4.6	
**36**	magnolol	VH	23.5 ± 5.7	4.3 ± 6.6	
**37**	oxypeucedanin hydrate	VH	15.7 ± 3.2	10.9 ± 9.0	
**38**	MolPort-001-633-809	VH	6.4 ± 4.6	3.0 ± 0.7	
**39**	Molport-000-428-993	VH	42.0 ± 1.3	10.0 ± 5.4	
**40**	Molport-001-540-175	VH	5.0 ± 2.6	1.6 ± 8.8	
**41**	Molport-002-532-107	VH	14.6 ± 13.1	9.6 ± 5.8	
**42**	Molport-005-910-551	VH	8.8 ± 7.0	4.89 ± 1.3	
**43**	Molport-002-520-481	VH	14.3 ± 3.4	10.0 ± 5.4	
**44**	Molport-007-627-328	VH	1.5 ± 1.2	6.4 ± 4.2	
**45**	Molport-002-535-048	VH	2.6 ± 0.1	9.0 ± 4.3	
**46**	Molport-007-806-805	VH	14.0 ± 3.4	12.7 ± 6.9	
**47**	Molport-039-338-319	VH	0.5 ± 0.8	8.8 ± 5.6	
**48**	Molport-019-909-691	VH	0.3 ± 0.5	8.2 ± 2.3	

Five out of the 19 VHs for M^pro^, namely, **12**–**16**, showed more than 60% M^pro^ inhibition at 20 μM. Compounds **12**–**16** were identified as potent inhibitors, with IC_50_ values of 7.24, 6.70, 13.85, 4.82, and 5.25 μM, respectively (Figure S2, Supporting Information). In addition, compounds **22** and **23** were determined as weak M^pro^ inhibitors with IC_50_ values of 35.2 and 33.5 μM, respectively. It should be noted that baicalein (**4**) was selected as a positive control. It was previously reported as an NP with strong inhibitory activity against M^pro^ (IC_50_ values of 5.16 and 0.98 μM).^17,30^ In our assay, however, **4** showed significant enzyme inhibition only at higher concentrations. An IC_50_ of 50.6 μM was determined, which is comparable to that reported by Hengphasatporn et al.^31^ One reason for the previously reported low IC_50_ values might be the absence of a reducing agent such as dithiothreitol (DTT) in the assay buffer. Compound **4** contains a catechol moiety, a common PAINS motif that is known to form unspecific covalent bonds to proteins, e.g., by binding to the thiol of a cysteine.^32^ Since M^pro^ is a cysteine protease, it is plausible that the reported strong M^pro^ inhibition by **4** is due to covalent binding to the cysteine in the active center. In the presence of a reducing agent (as performed in the present study), **4** showed only moderate M^pro^ inhibitory activity.

Experimental validation of the PL^pro^ VHs was initially performed with a commercial PL^pro^ kit and revealed four VHs (**30**, **35**, **37**, **42**) with significant enzyme inhibition at 20 μM. These compounds were tested further for their antiviral activity against SARS-CoV-2 in two different cell lines (Caco-2, Calu-3, Table S5, Supporting Information). Herein, only **35** and its congener **36** were identified as moderately acting antiviral compounds. It should be noted that the supplier of the PL^pro^ kit did not provide details on substrate identity and buffer composition in the documentation and neither on later request. Hence, another assay based on the cleavage of Z-RLRGG-AMC was used to validate the results, as shown in Table 1. None of the tested VHs showed an enzyme inhibition >60% at 20 μM in this assay. However, compound **30** was confirmed as a weak PL^pro^ inhibitor with an IC_50_ value of 24.3 μM (Figure S3, Supporting Information). Interestingly, **30** as well as its nonacetylated derivative, shikonin, were previously reported to inhibit M^pro^ with IC_50_ values of 3.8 and 1.6 μM, respectively.^33^ Ma and co-workers revealed that the reported M^pro^ inhibition of shikonins is an assay artifact.^34^ However, herein, it is the first time that **30** is reported as a PL^pro^ inhibitor by ruling out assay interference through the use of controls addressing compound aggregation, background fluorescence, and protein oxidation.

**Figure 1:**
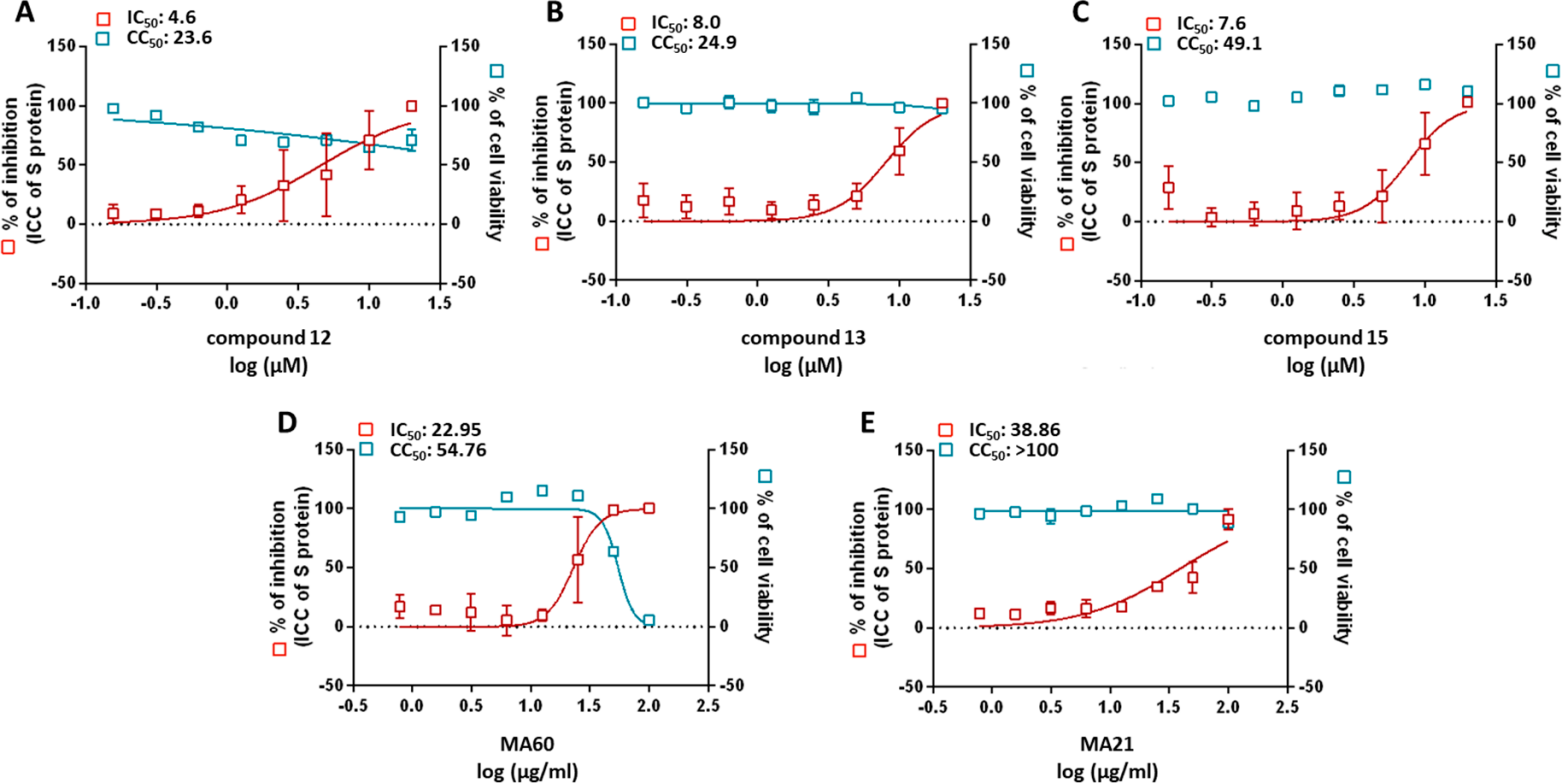
Dose–response curves showing anti-SARS-CoV-2 activity of compounds **12** (A), **13** (B), and **15** (C) and of the *Morus alba* root bark extracts MA60 (D) and MA21 (E) in Calu-3 cells, as determined by a spike staining assay.

To corroborate the enzymatic inhibition results, the five virtually identified and experimentally confirmed protease inhibitors **12**–**16**, as well as the determined weak inhibitors **22**, **23**, and **30**, were tested for antiviral activities against SARS-CoV-2 in Calu-3 cells. Compounds **12**, **13**, and **15** inhibited the SARS-CoV-2 replication significantly with IC_50_ values of 4.6, 8.0, and 7.6 μM, respectively (Table 3, Figure 1A–C). Although compounds **12**, **13**, and **15** showed some cytotoxicity at higher concentrations, they still exerted a decent selectivity toward SARS-CoV-2 with SIs of 5.1, 3.1, and 6.5, respectively.

**Table 3:** Evaluation of Anti-SARS-CoV-2 Activity and Cytotoxicity in Calu-3 Cells of Determined M^pro^ and PL^pro^ Inhibitors

compound	IC_50_ (μM)	CC_50_ (μM)	compound	IC_50_ (μM)	CC_50_ (μM)
remdesivir (**1**)	0.00125	>0.1	kuwanon L (**16**)	>20	97.7
sanggenon C (**12**)	4.6	23.6	kuwanon A (**22**)	35.6	47.7
sanggenon O (**13**)	8.0	24.9	morusin (**23**)	24.9	25.2
sanggenon D (**14**)	18.5	46.2	acetylshikonin (**30**)	>100	>100
sanggenon G (**15**)	7.6	49.1			

Compounds **12** to **16** are MDAAs, which are derived from the cycloaddition of a chalcone and a dehydroprenylphenol such as prenylated flavonoids.^35^ They are characteristic constituents of the root bark of the medicinal plant *Morus alba* L. (the white mulberry tree), traditionally used in Chinese medicine “sa̅ng bái pí”. In addition to testing the virtually predicted and isolated MDAAs, two extracts of the underlying herbal drug were prepared and also tested for their potential to inhibit the SARS-CoV-2 replication in Calu-3 cells. Thus, MA21 is a hydro-ethanolic extract with a content of 5.4% MDAAs, and MA60 represents an extract optimized toward a high yield of this bioactive compound class (29% MDAAs).^36^ For MA60 and MA21, IC_50_ values of 23.0 and 38.9 μg/mL were determined, respectively (Figure 1D,E).

### Docking Analysis and NMR Experiments

**Figure 2:**
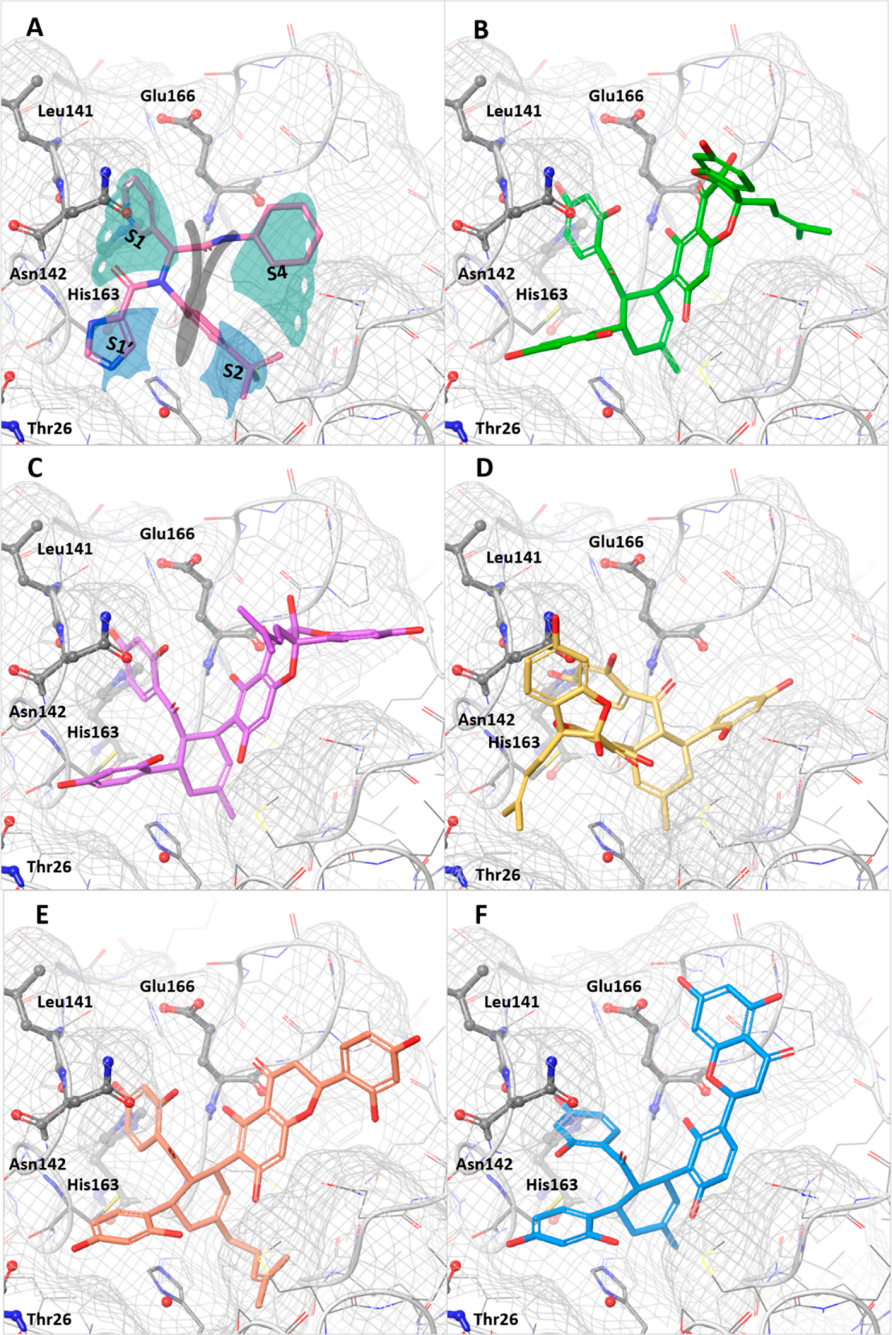
(A) Compound **8** (X77) bound to the butterfly-shaped substrate binding site of M^pro^ (PDB code: 6W63). (B–F) Docking poses of compounds **12** (green), **13** (purple), **14** (yellow), **15** (orange), and **16** (blue) in the binding site of 6W63. The substrate binding site is indicated as gray mesh. The water molecule is shown as a red sphere.

The substrate binding site of M^pro^ consists of four subsites, S1, S1′, S2, and S4 (Figure 2).^37^ These subsites form a “butterfly-shaped” binding pocket. Well-known M^pro^ inhibitors such as **8** (Figure 2A) occupy all of these subsites. Molecular docking suggests a similar occupation of all four subsites by **12**–**16** (Figure 2B–F). The conserved benzoyl and cyclohexene moieties of these compounds are placed in the S1 and the lipophilic S2 pocket, respectively, and align well with the pyridine and phenyl ring of **8**. For **12**, **13**, **15**, and **16**, the phenyl ring of the chalcone is embedded in S1′, while the flavonoid part occupies S3. In contrast, **14** shows an inverted binding orientation regarding the S1′ and S4 subsites in comparison to the other compounds. One reason could be the stereochemical (*R*)-configuration of **14** at position C-3″ (the connection between chalcone and flavonoid), which is opposite to the (*S*)*-*configuration of **12** and **13**. Similar observations were reported on cocrystallized human immunodeficiency virus (HIV) protease inhibitors.^38^ Regarding the suggested molecular interactions, all five MDAAs identified by docking could be predicted to form hydrogen bonds in S1 with Leu141, Asn142, and His163 and lipophilic interactions with residues forming S2. Additionally, all compounds except **14** were predicted to form hydrogen bonds with Thr26 and Glu166 in S1′ and S4, respectively. The hydrogen bonds to His163 and Glu166 and the lipophilic moiety in S2 were predicted to be also present for compound **8**. These interactions are considered to be essential for ligand binding.^39^

**Figure 3:**
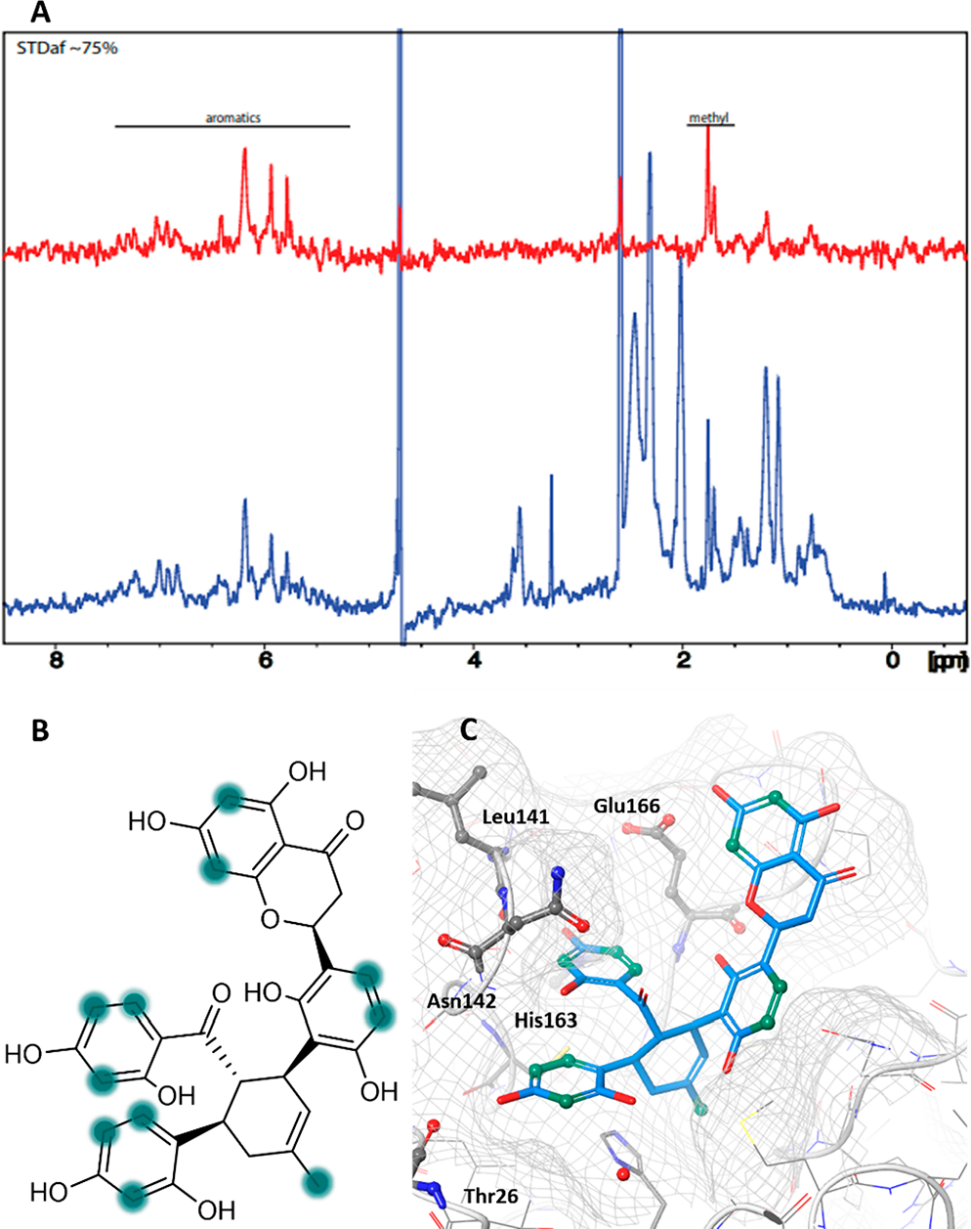
(A) STD NMR of M^pro^ and compound **16**. The STD difference spectrum is shown in red, with the STD off spectrum (1D) superposed in blue. (B) Proposed binding epitope map of **16**. (C) Combined presentation of the docking pose of **16** in 6W63. Determined interactions from STD NMR are highlighted in green (6B, 6C).

Validation of the ligand–target interactions for **12**–**16** was performed with STD NMR spectroscopy. The resulting STD NMR spectrum generated by the difference of recorded NMR spectra of ligand and protein with and without protein saturation (on- and off-resonance) indicates ligand protons likely to contact the protein. Hence, ligand moieties that are not involved in protein binding are likely to not show an STD signal. STD NMR experiments were conducted for each of the selected ligands in the presence of uniformly ^15^N-labeled recombinant M^pro^.^40^ An (^1^H, ^15^N)-HSQC of the purified protein was recorded for quality control and comparison with the previously reported protocol to ensure that the protein adopts the fully active dimeric form in vitro (Figure S4, Supporting Information).^41^ Only compounds **14** (Figure S5, Supporting Information) and **16** (Figure 3A) exhibited STD signals, while no STD signals were detected for **12**, **13**, and **15**, suggesting that these compounds bind to M^pro^ with a longer residence time in the binding site.^42^ The STD-AF is used for the quantitation of the response of the ligand in interaction with the receptor of the protein.^43^

The STD-AF of **14** and **16**, indicating how buried molecular moieties are in the binding site, were compared with the proposed binding poses from molecular docking (Figure 3B, Figure S5, Supporting Information). The relatively uniform STD-AF data indicate that compounds **14** and **16** exhibit contact with the protein involving all moieties, supporting the large interaction surface proposed by the docking studies (Figure 3C).

To confirm the binding of compounds **12**, **13**, and **15** to M^pro^, competitive relaxation dispersion experiments were carried out using compound **4** as a reporter ligand (Figures S6 and S7, Supporting Information). In the ^1^H Carr–Purcell–Meiboom–Gill relaxation dispersion (CPMG-RD) experiments, transverse relaxation rates of the ligand resonances are enhanced upon protein binding and therefore report on the bound population of the ligand.^44^ The addition of **12** or **15** to the mixture of **4** with M^pro^ reduced the relaxation rates of **4**, indicating its displacement from the binding site by **12** and **15** via competition (Figures S8 and S9, Supporting Information). Compound **13** however did not significantly displace **4**, suggesting a noncompetition, while both showed an enhanced transverse relaxation rate (Figure S9, Supporting Information).

In conclusion, MDAAs were discovered as a unique NP structural class with anti-SARS-CoV-2 activity. The results presented combine computational, biochemical, and biophysical approaches. The VHs sanggenon C (**12**), sanggenon O (**13**), and sanggenon G (**15**) were confirmed not only to inhibit the predicted target but also to exert potent phenotypic antiviral activity in Calu-3 cells with IC_50_ values of 4.6, 8.0, and 7.6 μM, respectively. Despite showing a slight cytotoxicity, compounds **12** and **15** have reasonable SI values of >5. In agreement with the applied virtual screening, compounds **12**–**16** act as ligands of M^pro^ and inhibit its activity at low micromolar concentrations. M^pro^ has been shown to be essential for viral replication. Hence, its inhibition by the MDAAs identified is a plausible antiviral mode of action. According to the predicted binding mode and the STD NMR data, MDAAs occupy four subsites in the substrate binding pocket. In addition, the results from the CPMG-RD experiments further support the assumed competitive M^pro^ inhibition of MDAAs by binding to the substrate binding site. The two resorcyl residues flank the catalytic Cys134 in the S1 and S1′ pockets, while the cyclohexene ring with the methyl (**12**, **13**) or prenyl (**15**) groups is accommodated in the lipophilic S2 pocket. The benzopyran moiety loosely covers the solvent-accessible S4 pocket. Since **12**, **13**, and **15** all share a similar docking pose with **16**, we propose the chalcone moiety as a promising starting point for improved potent M^pro^ inhibitors. These new findings further enrich the anti-infective and anti-inflammatory profile of the MDAAs investigated in this study. Previously, it has been shown that they exert (i) a pronounced in vitro anti-inflammatory activity,^36^ (ii) an antibacterial activity against *Staphylococcus aureus* and *Streptococcus pneumoniae* accompanied by a distinct inhibition of pneumococcal biofilm formation,^45^ (iii) an antiviral activity against different influenza virus strains, while (iv) being well tolerated by lung cells (A549, Calu-3).^36^ This was also confirmed for an MDAA-enriched extract (MA60) from mulberry root bark.^36^

From an unbiased chemical perspective, which was the starting point for the present in silico-driven, target-based drug discovery study, the obtained results underline the utility of NP virtual screening for the discovery of genuine NPs with high to moderate drug-likeness as novel anti-SARS-CoV-2 scaffolds. From a multitarget and multicomponent perspective, the rich bioactivity profile of the MDAAs derived from previous findings and from this study provide a rationale for the long-standing use of *M. alba* root bark “sa̅ng bái pí” in traditional Chinese medicine for the treatment of respiratory infections. It is worth mentioning that the root bark is the only plant part from white mulberry enriched with MDAAs. Commonly consumed mulberry fruits and other plant parts monographed in the Chinese pharmacopoeia such as leaves and twigs do not contain MDAAs studied in this work in detectable amounts.^36^ MDAAs and MDAA-enriched multicomponent extracts of *M. alba* root bark warrant further studies to evaluate their potential for the treatment of Covid-19 and other acute respiratory infections.

## Experimental Section

### Preparation of Molecular Databases for Molecular Docking

Two molecular databases were employed for docking: the MNPDB, which is a database of commercially available NPs and derivatives listed in catalogues of Molport chemical suppliers (https://www.molport.com),^25^ and the IHDB, which is a manually curated library of physically available compounds at the Division of Pharmacognosy of the Department of Pharmaceutical Sciences, University of Vienna.^24^ The databases were prepared for docking using RDKit and CDK nodes in KNIME.^46−48^ Entries with unconnected compounds were split with the RDKit salt stripper into individual entries.^46^ Next, the element filter of CDK was applied to filter any molecules containing C, H, N, O, P, S, I, Br, Cl, or F.^47^ The duplicate remover of LigandScout was employed to remove duplicates.^49,50^ Only molecules of high to moderate drug-likeness (MW of 130–900 Da; cLogP of −1.3–7.2; calculated with LigandScout) were included. Molecules with more than three undefined chiral centers were removed; those with undefined chiral centers were flagged. In the final step, one 3D conformation for each molecule was generated using Icon from LigandScout.^50,51^ For all molecules with an undefined stereocenter, all possible enantiomers were generated using LigPrep from Schrödinger.^52^ The prepared databases with 1,309 and 140,164 molecules were stored in MOL2 file format.

### Molecular Docking

For the selection of suitable target structures for docking, X-ray structures of M^pro^ and PL^pro^ from SARS-CoV and SARS-CoV-2 cocrystallized with noncovalent inhibitors bound to the substrate binding site and available from the Protein Data Bank (PDB, https://www.rcsb.org/) in October 2020 with a resolution <2.5 Å were considered. The electron density support of the key residues and ligands was verified in each structure, and structures with a concerning goodness of fit were rejected.^53,54^ Furthermore, the binding site alignment feature of the Schrödinger modeling suite was applied to select potentially different conceivable protein conformations and ligand–target interactions.^55^ Finally, one structure of M^pro^ (6W63) and two structures of PL^pro^ (7JN2, 4OW0) were selected for virtual screening. The PDB M^pro^ structure 6W63 contained two homodimer chains from which chain A was selected, as its ligand better fits the electron density map. The structures were prepared with the Protein Preparation Wizard of the Schrödinger modeling suite with default settings. The addition of hydrogens and the generation of heterostates were performed using Epik followed by the refinement of H-bond assignment.^56,57^ Water molecules beyond 5 Å from heteroatoms and fewer than two H-bonds to nonwaters were removed. In addition, all other solvents and ions were deleted. Finally, restrained minimization was performed using the OPLS3e force field.^58^

For virtual screening, the docking software GOLD (version 5.7.3) was employed.^23^ The docking runs were configured with the Docking Wizard of the GOLD software suite. For each ligand, the number of docking runs per molecule was set to 15. The fitness function ChemPLP was selected as the scoring function using default parameters. For the genetic algorithm parameters that determine docking speed, default settings were applied (“slow/automatic”). The reliability of the molecular docking procedure was first assessed by self-docking, which successfully retrieved docking poses of the corresponding ligands from the protein structures at RMSD values lower than 1 Å (Figures S8–S10, Table S6, Supporting Information). The docking results from both databases for all three protein structures were evaluated independently according to the following criteria: the top 100 ranked molecules from the IHDB, according to the ChemPLP score, were considered for visual inspection. For the analysis of the MNPDB results, an arbitrary threshold was set for each protein structure with regard to the ChemPLP score of the redocked ligands (80.00 for 7JN2 and 4OW0, 70.00 for 6W63). All docking poses above this threshold were then evaluated by visual inspection.

### Compounds and Chemicals

Compound **1** was obtained from Selleckchem (Houston, TX, USA). Compounds **4** (catalogue ID 465119), **7** (catalogue ID SML2961), **18** (catalogue ID C7243), **35** (catalogue ID H4914), and **36** (catalogue ID M3445) were obtained from Sigma-Aldrich (St. Louis, MO, USA). Compounds **5** (catalogue ID 15494869) and **6** (catalogue ID 15486559) were obtained from ThermoFisher. Compounds **11**–**16** were isolated from *Morus alba*.^45^ Compound **17** was isolated from the seeds of *Alpinia katsumadai*.^59^ Compound **19** (catalogue ID SRP04671c) was obtained from Sequoia Research Products, Ltd. (Pangbourne, UK). Compound **20** (catalogue ID PS020342) was obtained from Chengdu Push (Chengdu, People’s Republic of China). Compound **21** was previously isolated from *Sophora flavescens*. Compounds **22** (catalogue ID CFN90834), **23** (catalogue ID CFN97083), **24** (catalogue ID CFN97086), **25** (catalogue ID CFN92340), **26** (catalogue ID CFN99300), and **47** (catalogue ID CFN97728) were obtained from ChemFaces (Wuhan, People’s Republic of China). Compounds **27** (catalogue ID STK024437), **28** (catalogue ID STL521306), **29** (catalogue ID STK743870), **39** (catalogue ID STK798803), **40** (catalogue ID STK439583), **41** (catalogue ID STL515531), **42** (catalogue ID STL099426), **43** (catalogue ID STK923950), **45** (catalogue ID STL517522), and **48** (catalogue ID STL521306) were obtained from Vitas-M Laboratory (Wuhan, People’s Republic of China). Compound **30** (catalogue ID 018-19191) was obtained from Wako Pure Chemical Industries (Osaka, Japan). Compound **31** was obtained from Merck (Darmstadt, Germany). Compound **32** was obtained from Boehringer-Ingelheim (Ingelheim am Rhein, Germany). Compounds **33** and **34** were obtained from Madaus (Köln, Germany). Compound **37** was isolated from roots of *Peucedanum ostruthium*,^60^ and **38** (catalogue ID AK-968/41026577) was obtained from SPECS (Delft, Netherlands). **44** (catalogue ID C382-0112) and **46** (catalogue ID F671-0036) were obtained from ChemDiv (San Diego, CA, USA). The purity of compounds **12**–**16** was checked with UPLC-ELSD (Supporting Information). All compounds were prepared as 100 mM stock solutions in DMSO. Working stocks were diluted accordingly to achieve a final DMSO concentration of 0.2% in the experiments. For STD experiments, a 10 mM stock solution of compounds in *d*_6_-DMSO was prepared. All compound stocks were stored at −20 °C until use.

### Protein Production

The DNA sequence encoding M^pro^ SARS-CoV-2 (residues 1–306) was synthesized by GenScript (The Netherlands) with an N-terminal solubility tag and C-terminal affinity tag and cloned into a Pet-28a+ expression vector. The fusion protein encoded for glutathione *S*-transferase (GST) tag-tobacco etch virus (TEV) cleavage site–M^pro^–6xHis-tag was expressed in *E. coli* RIL cells and purified. The bacteria were grown at 37 °C in labeled ^15^N-M9 medium supplemented with kanamycin and chloramphenicol. When A_600 nm_ reached ∼0.8, the temperature was lowered to 30 °C and induced with 1 mM isopropyl β-d-1-thiogalactopyranoside (IPTG) and left overnight. The solubility tag was self-cleaved after expression, and cells were harvested in Linx6000 at 4 °C with a speed of 4000*g*. The cell lysate was passed through a HisTrap HP column (Cytivia). Then, TEV protease, produced in-house, was added to the fractions containing this protein, and the mixture was dialyzed overnight at 4 °C, followed by another pass through the HisTrap HP column. The relevant fractions were concentrated and applied to a Sephadex G75 gel filtration column (Cytivia), as reported.^41^ The purified SARS-CoV-2 M^pro^ protein quality check was assessed from the 2D-[^1^H, ^15^N]-TROSY-HSQC spectrum (Figure S5). The protein was stable at room temperature for the time of the NMR experiments. Unlabeled M^pro^ was produced following the same protocol using Lysogeny broth medium instead of M9 medium.

### M^pro^ Assay

Unlabeled M^pro^ was buffer exchanged to 50 mM Tris-HCl (pH 7.55), 1 mM EDTA, and 5 mM DTT. VHs that showed inhibitory activity at 20 μM were selected for further characterization. Enzyme activity measurements were performed in buffer (50 mM Tris-HCl (pH 7.55), 1 mM EDTA, and 5 mM DTT) at 30 °C. Mixtures of 1 μM M^pro^ with a series of increasing compound concentrations (0–300 μM) were preincubated for 30 min at 30 °C in black flat-bottomed 96-well plates (Costar #3915). Then, 10 μM of the fluorescently labeled substrate (crb1101508j, Discovery Peptides, Billingham, UK), containing an N-terminal 5-carboxyfluorescein (5-FAM) and the fluorescence quencher dabcyl, was added. Upon cleavage of the peptide by SARS-CoV-2 M^pro^, the 5-FAM group and the dabcyl quencher are separated, and a fluorescence signal can be detected. Fluorescence signals were obtained by exciting the dye at 483 nm and reading at 530 nm using a Tecan M200 plate reader. Data were measured for 60 cycles every 30 s at 30 °C and analyzed using Rstudio.^61^ For each data point, measurements were replicated five times.

### Recording of NMR Data

Isotopically ^15^N-labeled M^pro^ was buffer exchanged to 10 mM sodium phosphate buffer, pH 7.5 in D_2_O using a concentrator. A 10 mM stock solution in *d*_6_-DMSO was prepared for each compound. The samples were prepared with a ratio of 1:25 protein:ligand. Each tube contained 2 μM of protein in deuterated buffer containing 10 mM sodium phosphate pH 7.5, with 50 μM of ligand for a final volume of 500 μL in a 5 mm tube.

All experiments were recorded at 298 K on a 500 MHz Bruker Advance spectrometer equipped with a prodigy 5 mm TCI-cryoprobe and using TopSpin 3.1. All spectra were analyzed in Bruker TopSpin software versions 3.1 and 4.1.1. Compound structures were drawn by Chemdraw.^62^

The ^1^H 1D proton experiments for the ligands were obtained with 1024 scans, 8k points, and an acquisition time of 0.51 s for a total experimental time of approximately 20 min. The STD experiments were acquired with 1024 scans, 8k points, and an interscan delay of 2.5 s, with an acquisition time of 0.59 s. The protein saturation was performed with a train of 50 ms Gaussian-shaped pulses, centered at −1 ppm, for a total of 2.5 s saturation time. The protein signals were filtered out using a spin-lock period of 30 ms and field strength of 10 kHz. The total duration of each STD experiment was approximately 90 min. An STD hit was considered when the STD NMR signals had a signal-to-noise ratio of min 6.5. Control experiments were repeated without the protein. Epitope mapping was determined by calculating the STD amplification factors.^43^ The ^1^H CPMG-RD experiments were conducted with relaxation delays set to 0, 8, 120, 200, and 400 ms.^63^ The samples were prepared with same conditions as in the STD NMR experiments. The CPMG-RD experiments used an interscan relaxation delay of 2.5 s, 256 scans, and an acquisition time of 2.04 s with 8k points. The intensities were fitted to a monoexpansional decay using Rstudio.^61^

### PL^pro^ Assay

VHs were tested for PL^pro^ inhibition using a fluorescence assay based on cleavage of a Z-RLRGG-AMC substrate (abcr; catalogue ID AB478009). The assay buffer contained 40 mM Tris-HCl (pH 7.8), 100 mM NaCl, 5 mM DTT, 0.01% (w/v) Triton X 100, and 0.1 mg/mL BSA. Compounds were preincubated with His-PL^pro^ (BPS-100735-1, BPS Biosciences) for 60 min. The substrate was added, and fluorescence at Exc/Emi 360/460 nm was measured every 3 min for 72 min using a Tecan Sparks plate reader (Männedorf, Switzerland). The final concentrations of PL^pro^ enzyme and substrate were 50 nM and 50 μM, respectively. The assay volume was 100 μL. Final test concentrations of VHs were 20 and 100 μM with 0.2% DMSO in assay buffer. Positive control **7** was tested at a final concentration of 10 μM and 0.2% DMSO in assay buffer. Experiments were performed in two well replicates. Two background control wells without enzymes were measured in parallel to correct for background fluorescence. Means of three independent experiments are presented. PL^pro^ inhibition curves were obtained by measuring the enzymatic activity at six compound concentrations. The IC_50_ values of the compounds were determined by plotting enzyme inhibition against various concentrations of the test inhibitor by using the dose–response curve in GraphPad Prism 6.^64^

### Cells and Viruses

Calu-3 cells (ATCC, Manassas, VA, USA) were grown at 37 °C in minimal essential medium (MEM) supplemented with 10% fetal bovine serum (FBS), 100 IU/mL of penicillin, and 100 μg/mL of streptomycin. All culture reagents were purchased from Sigma-Aldrich. SARS-CoV-2 was isolated by using the Caco-2 clonal subline as previously described.^65^ SARS-CoV-2 isolate (SARS-CoV-2/FFM7, MT358643) used in the experiment had undergone a maximum of three passages, and stocks were stored at −80 °C.

### Antiviral Assay

Confluent layers of cells in 96-well plates were treated with decreasing concentrations of each test compound and subsequently infected with SARS-CoV-2 at a MOI of 0.01. Evaluation of inhibitory rate was performed by immunohistochemistry of viral spike protein 48 h postinfection. Briefly, cells were fixed with acetone–methanol (40:60) solution, and immunostaining was performed using a monoclonal antibody directed against the spike protein of SARS-CoV-2 (1:1500, Sinobiological), which was detected with a peroxidase-conjugated anti-rabbit secondary antibody (1:1,000, Dianova), followed by addition of AEC substrate. The spike positive area was scanned and quantified using a Bioreader 7000-F-Z-I microplate reader (Biosys). The results are expressed as percentage of inhibition relative to a virus control that received no test compound.

## Supporting Information Available

Additional figures showing structural differences in the active site between PDB structures 7JN2 and 4OW0, concentration–response curves from M^pro^ and PL^pro^ assay, STD NMR data of compound **14**, results from CPMG-RD experiments, results from redocking studies; supporting tables containing virtual hits and additional in vitro antiviral activities for VHs of PL^pro^; UPLC analyses of compounds **12**–**16** (PDF)
