# Supplementary material for: Identification of Natural Products Inhibiting SARS-CoV-2 by Targeting Viral Proteases: A Combined in Silico and in Vitro Approach
Source: J Nat Prod. 2023 Jan 18;86(2):264–75. doi: 10.1021/acs.jnatprod.2c00843 (PMC9885530; doi:10.1021/acs.jnatprod.2c00843)

Supporting information

Identification of Natural Products Inhibiting SARS-CoV-2 by Targeting the Viral Proteases: A Combined in Silico and in Vitro Approach

Andreas Wasilewicz,[†,‡,#] Benjamin Kirchweger,[†,#] Denisa Bojkova,[§] Marie Jose Abi Saad,[†,‡] Julia Langeder,[†,‡] Matthias Bütikofer,[⊥] Sigrid Adelsberger,[†,‡] Ulrike Grienke,[†] Jindrich Cinatl Jr,[§] Olivier Petermann,[ǀ,○] Leonardo Scapozza,[ǀ,○] Julien Orts,[†] Johannes Kirchmair,[†] Holger F. Rabenau,[§] Judith M. Rollinger,[†,*]

[†] Department of Pharmaceutical Sciences, Faculty of Life Sciences, University of Vienna, Josef-Holaubek-Platz 2, 1090 Vienna, Austria

[‡] Vienna Doctoral School of Pharmaceutical, Nutritional, Sport Sciences, University of Vienna, Josef-Holaubek-Platz-2, 1090 Vienna, Austria

[§] Institute of Medical Virology, University Hospital Frankfurt, Paul-Ehrlich-Straße 40, 60596 Frankfurt am Main, Germany

[⊥] Swiss Federal Institute of Technology, Laboratory of Physical Chemistry, ETH Zurich, Vladimir-Prelog-Weg 1-5/10 8093 Zurich, Switzerland

[ǀ] Pharmaceutical Biochemistry Group, School of Pharmaceutical Sciences, University of Geneva, Switzerland

[○] Institute of Pharmaceutical Sciences of Western Switzerland, University of Geneva, Switzerland

Table of content

Figure S1. Superimposition of substrate-binding sites of PL^{pro} structures 7JN2 and 4OW0..... 3

Table S1. Distribution of virtual hits across protein structures.. 3

Table S2. Predicted virtual hits for M^{pro} structure 6W63... 4

Table S3. Predicted virtual hits for PL^{pro} structure 7JN2... 6

Table S4. Predicted virtual hits for PL^{pro} structure 4OW0... 8

Figure S2. Concentration-response curves of compounds **12-16** against M^{pro} 9

Table S5. Inhibitory activities of virtual hits against PL^{pro} 10

Figure S3. Concentration-response curves of compounds **7** and **30** against PL^{pro}.................. 11

Figure S4. 2D-[1H, 15N]-TROSY-HSQC of M^{pro}... 12

Figure S5. STD NMR of M^{pro} with compound **14**... 12

Figure S6. Competition CPMG experiments 13

Figure S7. CPMG relaxation decay curves.. 14

Figure S8. Re-docking of co-crystallized ligand of 6W63.. 15

Figure S9. Re-docking of co-crystallized ligand of 7JN2... 15

Figure S10. Re-docking of co-crystallized ligand of 4OW0... 16

Table S6. RMSD values of re-docked ligands of 6W63, 7JN2 and 4OW0............................ 16

UPLC analysis of compounds **12-16**... 17

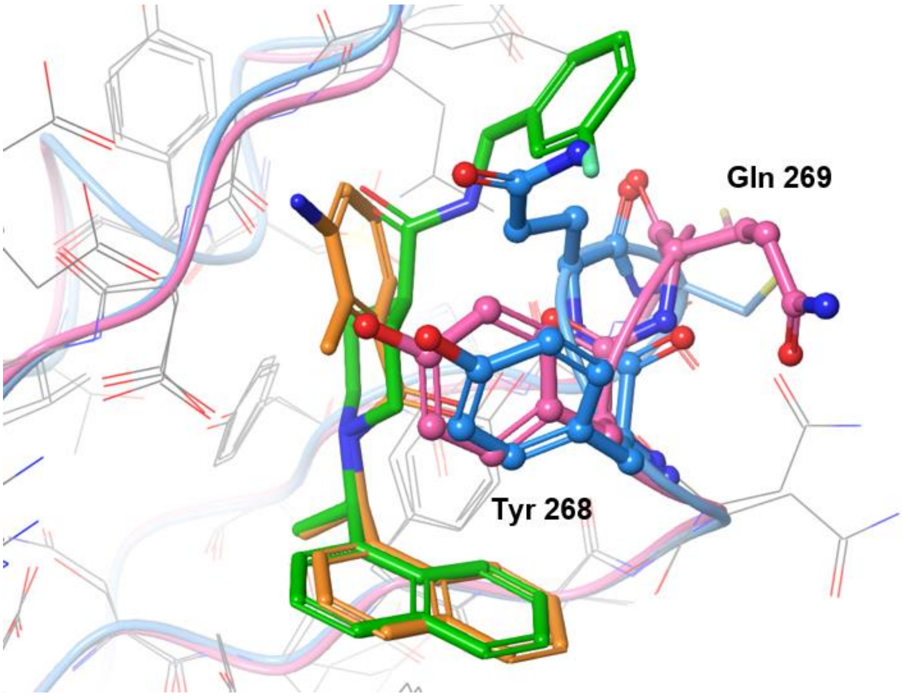

Figure S1. Superimposition of the subtrate-binding sites of PL^{pro} structures 7JN2 (blue) and 4OW0 (pink) and their respective co-crystallized ligands Y41 (orange) and S88 (green).

Table S1. Distribution of virtual hits across protein structures

		Virtual Hits		
Protease	PDB code	IHDB	MNPDB	In Total
M^{pro}/3CL^{pro}	6W63	12	13	25
PL^{pro}	4OW0	5	6	11
	7JN2	13	12	25
				**61**

Table S2. Predicted virtual hits from M^{pro} structure 6W63 ranked by ChemPLP score. Tested hits are presented in bold.

compound name	CAS number	SMILES code	ChemPLP score	database origin	HitDexter 2.0	FAF Drugs 4
**15, Sanggenon G**	**85698-31-3**	**CC(C)=CCCC1=C[C@@H]([C@H]([C@@H](C1)C1=C(O)C=C(O)C=C1)C(=O)C1=CC=C(O)C=C1O)C1=C(O)C2=C(O[C@@H](CC2=O)C2=C(O)C=C(O)C=C2)C=C1O**	**89,47**	**INDB**	**Non-promiscous in CDRA, promiscous in PSA**	**Accepted**
MolPort-005-952-021		C[C@@]1(CC[C@]2([C@@H](C1)C(C[C@@H]2NC(=O)C3=CC=CC=C3)(C)C)CCC(=O)NCC4=CN=CC=C4)O	86,13	MNPDB	Non-promiscous in PSA and CDRA	Accepted
5,7,2',4'-tetrahydroxy-3-geranylflavone	376361-87-4	CC(=CCC/C(=C/CC1=C(OC2=CC(=CC(=C2C1=O)O)O)C3=C(C=C(C=C3)O)O)/C)C	81,4742	MNPDB	Promiscous in PSA and CDRA	Accepted
**29, MolPort-002-206-761**		**CN1C2=C(C(=O)N(C1=O)C)N(C(=N2)SCC(=O)NC3=CC4=C(C=C3)OCO4)CC5=CC(=CC=C5)Cl**	**80,98**	**MNPDB**	**Non-promiscous in PSA and CDRA**	**Intermediate (halogenure)**
**25, Ampelopsin F**	**151487-08-0**	**C1=CC(=CC=C1[C@@H]2[C@H]3[C@@H](C4=C([C@@H]2C5=C3C=C(C=C5O)O)C=C(C=C4O)O)C6=CC=C(C=C6)O)O**	**80,84**	**MNPDB**	**Promiscous in PSA and CDRA**	**Intermediate (phenol)**
**11, Sanggenol A**	**174423-30-4**	**CC(=CCC/C(=C/CC1=C(C=CC(=C1O)[C@@H]2CC(=O)C3=C(C=C(C=C3O2)O)O)O)/C)C**	**80,74**	**INDB**	**Promiscous in PSA and CDRA**	**Accepted**
**28, MolPort-035-701-904**		**C1=CC=C2C(=C1)C3N(C(=O)C4=CC=CC=C4N3C2=O)CCCC(=O)NCC5=CN=CN5**	**80,08**	**MNPDB**	**Non-promiscous in PSA and CDRA**	**Accepted**
**27, MolPort-000-689-498**		**COC1=CC=C(C=C1)C(=O)N(CCN(CC2=CC=CC=C2)C(=O)C3=CC=C(C=C3)OC)CC4=CC=CC=C4**	**77,31**	**MNPDB**	**Non-promiscous in PSA and CDRA**	**Accepted**
**26, Sarcandrone B**	**1190225-48-9**	**COC1=C(C(=C(C(=C1)O)C(=O)/C=C/C2=CC=CC=C2)O)[C@H]3C[C@@H](OC4=C(C(=CC(=C34)OC)O)OC)C5=CC=CC=C5**	**77,23**	**MNPDB**	**Non-promiscous in PSA and CDRA**	**Rejected (covalent binder)**
**12, Sanggenon C**	**80651-76-9**	**CC1=C[C@@H]([C@@H]([C@H](C1)C2=C(C=C(C=C2)O)O)C(=O)C3=C(C=C(C=C3)O)O)C4=C(C5=C(C=C4O)O[C@@]6(C7=C(C=C(C=C7)O)O[C@@]6(C5=O)O)CC=C(C)C)O**	**76,61**	**INDB**	**Non-promiscous in CDRA, promiscous in PSA**	**Accepted**
**14, Sanggenon D**		**CC(C)=CCC12OC3=C(C(O)=C([C@@H]4C=C(C)C[C@@H]([C@H]4C(=O)C4=CC=C(O)C=C4O)C4=CC=C(O)C=C4O)C(O)=C3)C(=O)C1(O)OC1=C2C=CC(O)=C1**	**76,26**	**INDB**	**Non-promiscous in CDRA, promiscous in PSA**	**Accepted**
MolPort-000-717-985	672272-01-4	CN1C=C(C2=CC=CC=C21)/C=C(/C(=O)NCCCO)\NC(=O)C3=CC(=C(C=C3)OC)OC	75,77	MNPDB	Non-promiscous in PSA and CDRA	Rejected (covalent binder)
Lupulone	468-28-0	CC(C)CC(=O)C1=C(C(=C(C(C1=O)(CC=C(C)C)CC=C(C)C)O)CC=C(C)C)O	75,35	INDB	Non-promiscous in PSA and CDRA	Rejected (covalent binder)
**24, Morusinol**	**62949-93-3**	**CC1(C=CC2=C(O1)C=C(C3=C2OC(=C(C3=O)CCC(C)(C)O)C4=C(C=C(C=C4)O)O)O)C**	**75,03**	**MNPDB**	**Non-promiscous in PSA and CDRA**	**Accepted**
8-Isomulberrin hydrate	1432063-35-8	CC1(CCC2=C(O1)C=C(C3=C2OC(=C(C3=O)CCC(C)(C)O)C4=C(C=C(C=C4)O)O)O)C	74,25	MNPDB	Non-promiscous in PSA and CDRA	Accepted
**22, Kuwanon A**	**62949-77-3**	**CC(=CCC1=C(OC2=CC(=CC(=C2C1=O)O)O)C3=C4C(=C(C=C3)O)C=CC(O4)(C)C)C**	**74,05**	**MNPDB**	**Promiscous in PSA and CDRA**	**Accepted**
Acrovestone	24177-16-0	CC(C)CC(C1=C(C(=C(C(=C1O)CC=C(C)C)O)C(=O)C)O)C2=C(C(=C(C(=C2O)CC=C(C)C)OC)C(=O)C)O	74,02	INDB	Non-promiscous in PSA and CDRA	Accepted
**18, Chicoric acid**	**6537-80-0**	**C1=CC(=C(C=C1/C=C/C(=O)O[C@@H](C(=O)O)[C@@H](OC(=O)/C=C/C2=CC(=C(C=C2)O)O)C(=O)O)O)O**	**72,68**	**INDB**	**Non-promiscous in PSA and CDRA**	**Rejected (1 PAINS, covalent binder)**
**23, Morusin**	**62596-29-6**	**CC(=CCC1=C(OC2=C(C1=O)C(=CC3=C2C=CC(O3)(C)C)O)C4=C(C=C(C=C4)O)O)C**	**72,62**	**MNPDB**	**Promiscous in PSA and CDRA**	**Accepted**
**21, Kurarinone**	**34981-26-5**	**CC(=CC[C@H](CC1=C2C(=C(C=C1O)OC)C(=O)C[C@H](O2)C3=C(C=C(C=C3)O)O)C(=C)C)C**	**73,32**	**INDB**	**Non-promiscous in PSA and CDRA**	**Intermediate (halo alkene)**

**16, Kuwanon L**	**88524-65-6**	**CC1=C[C@@H]([C@H]([C@@H](C1)C2=C(C=C(C=C2)O)O)C(=O)C3=C(C=C(C=C3)O)O)C4=C(C=CC(=C4O)[C@@H]5CC(=O)C6=C(C=C(C=C6O5)O)O)O**	**72,28**	**INDB**	**Non-promiscous in PSA and CDRA**	**Accepted**
**13, Sanggenon O**	**101664-32-8**	**CC1=C[C@@H]([C@@H]([C@H](C1)C2=C(C=C(C=C2)O)O)C(=O)C3=C(C=C(C=C3)O)O)C4=C(C5=C(C=C4O)O[C@]6(C7=C(C=C(C=C7)O)O[C@]6(C5=O)O)CC=C(C)C)O**	**69,51**	**INDB**	**Non-promiscous in CDRA, promiscous in PSA**	**Accepted**
**19, Cynarine**	**30964-13-7**	**C1[C@H]([C@@H]([C@@H](C[C@]1(C(=O)O)OC(=O)/C=C/C2=CC(=C(C=C2)O)O)OC(=O)/C=C/C3=CC(=C(C=C3)O)O)O)O**	**67,73**	**INDB**	**Non-promiscous in PSA, promiscous in CDRA**	**Rejected (1 PAINS, covalent binder)**
**17, Katsumadain A**	**252239-83-1**	**C1[C@H](OC2=C([C@@H]1C3=CC=CC=C3)C(=O)OC(=C2)/C=C/C4=CC=CC=C4)CC(=O)CCC5=CC=CC=C5**	**63,64**	**INDB**	**Non-promiscous in PSA and CDRA**	**Rejected (aliphatic ketone)**
**20, Neobavaiso-flavone**	**41060-15-5**	**CC(=CCC1=C(C=CC(=C1)C2=COC3=C(C2=O)C=CC(=C3)O)O)C**	**58,68**	**INDB**	**Promiscous in PSA and CDRA**	**Accepted**

INDB, in-house database; MNPDB, Molport Natural Product Database; PSA, primary screening assay; CDRA, confirmatory dose-response asssay

Table S3. Predicted virtual hits from PL^{pro} structure 7JN2 ranked by ChemPLP score. Tested hits are presented in bold.

compound name	CAS number	SMILES code	ChemPLP score	database origin	HitDexter 2.0	FAF Drugs 4
MolPort-000-851-002		CCOC(=O)CCC1=C(C2=C(C(=C(C=C2)O)C[NH2+]CC3=CC(=C(C=C3)OC)OC)OC1=O)C.[Cl-]	118,84	MNPDB	Non-promiscous in PSA and CDRA	Intermediate (1 PAINS)
**48, MolPort-019-909-691**	**1324072-44-7**	**CC(C)CCN1C=C(C2=CC=CC=C2C1=O)C(=O)NCC3=CC(=C(C=C3)OC)OC**	**102,79**	**MNPDB**	**Non-promiscous in PSA and CDRA**	**Accepted**
**39, MolPort-000-428-993**		**COCCN1C(C(C(=O)C2=CC=C(OC)C(C)=C2)=C(O)C1=O)C1=CC=C(O)C=C1**	**97,68**	**MNPDB**	**Non-promiscous in PSA and CDRA**	**Rejected (covalent binder)**
Molport-001-013-844		C1=CC=C(C=C1)OC2=CC=C(C=C2)C(=O)NCC3=CC=CC4=CC=CC=C43	97,68	MNPDB	Non-promiscous in PSA and CDRA	Accepted
**44, MolPort-007-627-328**		**COC1=CC=C(C=C1)S(=O)(=O)NCC2=CC=C(C=C2)C(=O)NCC3=CC=CC=C3**	**97,00**	**MNPDB**	**Non-promiscous in PSA and CDRA**	**Accepted**
Molport-002-826-944		CC(C1=CC(=CC=C1)C(=O)C2=CC=CC=C2)C(=O)OC3=CC=CC4=CC=CC=C43	96,46	MNPDB	Non-promiscous in PSA and CDRA	Accepted
**47, MolPort-039-338-319**	**53505-68-3**	**COC1=C(C=CC(=C1)CCCO)OC(CC2=CC(=C(C=C2)O)OC)CO**	**96,28**	**MNPDB**	**Non-promiscous in PSA and CDRA**	**Accepted**
MolPort-000-665-142		CCCCCCC1=CC(=C(C=C1O)O)C(=O)COC2=CC(=CC(=C2)C)C	94,98	MNPDB	Non-promiscous in PSA and CDRA	Rejected (conecutive alkyl chains)
**42, MolPort-005-910-551**	**951970-62-0**	**CC(CC1=CNC2=CC=CC=C21)NC(=O)C3=CC=C(C=C3)OC4=COC5=C(C4=O)C=CC(=C5)OC**	**93,61**	**MNPDB**	**Non-promiscous in PSA and CDRA**	**Accepted**
**40, MolPort-001-540-175**		**C1=CC=C(C=C1)CCCNC(=O)CC(C2=CC=CC=C2)C3=CC=CC=C3**	**93,49**	**MNPDB**	**Non-promiscous in PSA and CDRA**	**Accepted**
**45, Molport-002-535-048**	**865281-26-1**	**CC1=C(C(=CC=C1)C(C)C)NC(=O)CC2=C(C3=C(C(=C(C=C3)OC)OC)OC2=O)C**	**91,58**	**MNPDB**	**Non-promiscous in PSA and CDRA**	**Intermediate (Coumarines)**
**31, Capsaicin**	**404-86-4**	**CC(C)/C=C/CCCCC(=O)NCC1=CC(=C(C=C1)O)OC**	**86,70**	**IHDB**	**Non-promiscous in PSA, promiscous in CDRA**	**Accepted**
**30, Acetylshikonin**	**24502-78-1**	**CC(=CC[C@H](C1=CC(=O)C2=C(C=CC(=C2C1=O)O)O)OC(=O)C)C**	**78,92**	**IHDB**	**Non-promiscous in PSA and CDRA**	**Rejected (3 PAINS, covalent binder**
**34, Silibinin**		**COC1=C(O)C=CC(=C1)C1OC2=C(OC1CO)C=CC(=C2)[C@H]1OC2=CC(O)=CC(O)=C2C(=O)[C@@H]1O**	**78,50**	**IHDB**	**Promiscous in PSA and CDRA**	**Accepted**
**33, Silicristin**	**33889-69-9**	**COC1=C(C=CC(=C1)[C@H]2[C@@H](C3=C(O2)C(=CC(=C3)[C@@H]4[C@H](C(=O)C5=C(C=C(C=C5O4)O)O)O)CO)O**	**77,85**	**IHDB**	**Promiscous in PSA and CDRA**	**Accepted**
**38, 001-633-809**		**CN1C=C(C=N1)CNC(=O)C2=C(C=CC(=C2)[N+](=O)[O-])Cl**	**77,15**	**IHDB**	**Non-promiscous in PSA and CDRA**	**Intermediate (nitro)**
**32, Lobeline**	**90-69-7**	**CN1[C@@H](CCC[C@@H]1CC(=O)C2=CC=CC=C2)C[C@@H](C3=CC=CC=C3)O**	**72,77**	**IHDB**	**Non-promiscous in PSA and CDRA**	**Accepted**
MolPort-003-292-157		COC1=CC2=C(C=C1)C(=CC(=O)O2)COC(=O)C3=CC=CC=C3NCCO	93,62	MNPDB	Non-promiscous in PSA and CDRA	Intermediate (coumarines)
MolPort-009-375-739	1110928-66-9	CC1=CC(=C(C=C1)OC)C(=O)OC(C)C(=O)C2=C(NC3=CC=CC=C32)C	91,20	MNPDB	Non-promiscous in PSA and CDRA	Accepted
Isovalerylshikonin	52387-14-1	CC(C)CC(=O)O[C@H](CC=C(C)C)C1=CC(=O)C2=C(C=CC(=C2C1=O)O)O	85,57	IHDB	Non-promiscous in PSA and CDRA	Rejected (3 PAINS, covalent binder)
Arctiin	20362-31-6	COC1=C(C=C(C=C1)C[C@H]2COC(=O)[C@@H]2CC3=CC(=C(C=C3)O[C@H]4[C@@H]([C@H]([C@@H]([C@H](O4)CO)O)O)O)OC)OC	84,03	IHDB	Non-promiscous in PSA and CDRA	Accepted
β-β-Dimethylacryl-shikonin	24502-79-2	CC(=CC[C@H](C1=CC(=O)C2=C(C=CC(=C2C1=O)O)O)OC(=O)C=C(C)C)C	83,47	IHDB	Non-promiscous in PSA and CDRA	Rejected (3 PAINS, covalent binder)

5-Geranyloxy-7-methoxycoumarin	7380-39-4	CC(=CCC/C(=C/COC1=CC(=CC2=C1C=CC(=O)O2)OC)/C)C	81,90	IHDB	Non-promiscous in PSA and CDRA	Intermediate (coumarines)
Bergamottin	7380-40-7	CC(=CCC/C(=C/COC1=C2C=CC(=O)OC2=CC3=C1C=CO3)/C)C	75,33	IHDB	Non-promiscous in PSA and CDRA	Intermediate (coumarines, furanocoumarines)
Ostruthol	642-08-0	C/C=C(/C)\C(=O)O[C@H](COC1=C2C=CC(=O)OC2=CC3=C1C=CO3)C(C)(C)O	73,90	IHDB	Non-promiscous in PSA and CDRA	Rejected (covalent binder)

INDB, in-house database; MNPDB, Molport Natural Product Database; PSA, primary screening assay; CDRA, confirmatory dose-response assay

Table S4. Predicted virtual hits from PL^{pro} structure 4OW0 ranked by ChemPLP score. Tested hits are presented in bold.

compound name	CAS number	SMILES code	ChemPLP score	database origin	HitDexter 2.0	FAF Drugs 4
MolPort-047-538-149		C1CC2=C(C3=CC=CC=C3N=C2C1)NC(=O)C[NH2+]CCC4=CC=C(C=C4)O	95,80	MNPDB	Non-promiscous in PSA and CDRA	Intermediate (phenol)
MolPort-046-848-556	1030021-10-3	Cl.COC(=O)Cc1c(C)c2cc(CNCCc3ccccc3)c(O)c(C)c2oc1=O	95,48	MNPDB	Non-promiscous in PSA and CDRA	Intermediate (1 PAINS, coumarines)
Molport-010-696-387		CCN1C2=CC=CC=C2N(C(=O)C1=O)CC3=CC=C(C=C3)C(=O)NCC4=CC=C(C=C4)OC	94,46	MNPDB	Non-promiscous in PSA and CDRA	Intermediate (1 PAINS)
**46, MolPort-007-806-805**		**CC1=NC2=CC=CC=C2N(C1=O)CC3=CC=C(C=C3)C(=O)NCC4=CC=C(C=C4)OC**	**91,27**	**MNPDB**	**Non-promiscous in PSA and CDRA**	**Accepted**
**41, MolPort-002-532-107**	**864760-73-6**	**COC1=C(C=C\2C(=C1)CCN/C2=C\C(=O)C3=CC=C(C=C3)CSC)OC**	**88,93**	**MNPDB**	**Non-promiscous in PSA and CDRA**	**Rejected (frequent hitter_dopamine, covalent binder)**
**43, MolPort-002-520-481**	**858762-81-9**	**COC1=CC=C(C=C1)C(=O)COC2=CC3=C(C=C2)C(=O)/C(=C/C4=C(C=CC(=C4)OC)OC)/O3**	**88,69**	**MNPDB**	**Promiscous in PSA and CDRA**	**Rejected (covalent binder)**
Byakangelicin	482-25-7	CC(C)([C@@H](COC1=C2C(=C(C3=C1OC(=O)C=C3)OC)C=CO2)O)O	84,10	IHDB	Non-promiscous in PSA and CDRA	Intermediate (coumarines, furanocoumarines)
**37, Oxypeucedanin hydrate**	**2643-85-8**	**CC(C)([C@@H](COC1=C2C=CC(=O)OC2=CC3=C1C=CO3)O)O**	**81,78**	**IHDB**	**Non-promiscous in PSA and CDRA**	**Intermediate (coumarines, furanocoumarines)**
Molport-000-699-830	5667-50-5	C1CN(CCN1C2=C(C=C(C=C2)[N+](=O)[O-])Cl)C(=O)C3=CC(=C(C=C3Cl)F)F	78,86	IHDB	Non-promiscous in PSA and CDRA	Intermediate (1 PAINS, nitro)
**36, Magnolol**	**528-43-8**	**C=CCC1=CC(=C(C=C1)O)C2=C(C=CC(=C2)CC=C)O**	**78,03**	**IHDB**	**Promiscous in PSA and CDRA**	**Intermediate (Halo alkene)**
**35, Honokiol**	**35354-74-6**	**C=CCC1=CC(=C(C=C1)O)C2=CC(=C(C=C2)O)CC=C**	**70,49**	**IHDB**	**Promiscous in PSA and CDRA**	**Intermediate (Halo alkene)**

INDB, in-house database; MNPDB, Molport Natural Product Database; PSA, primary screening assay; CDRA, confirmatory dose-response assay

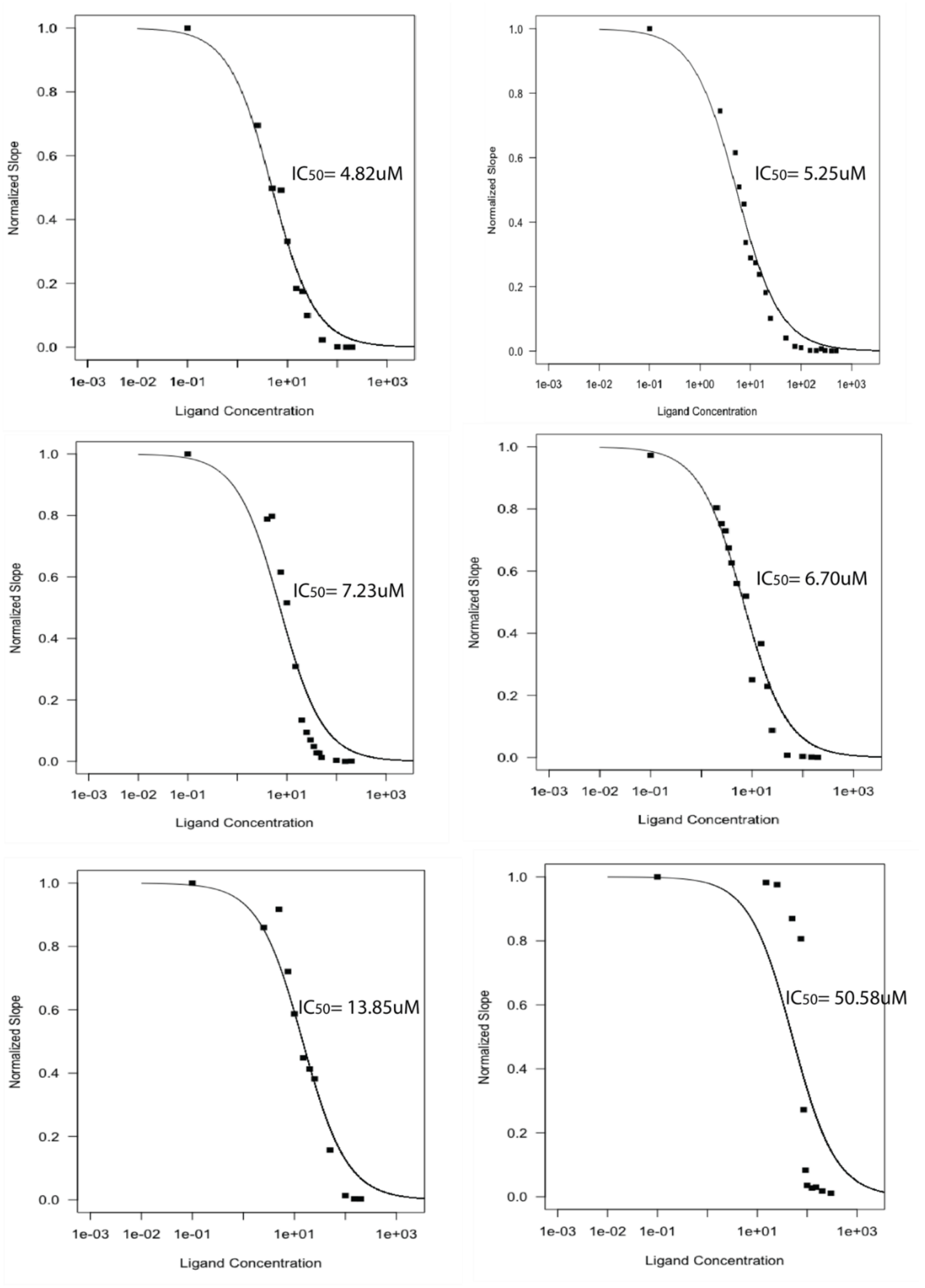

Figure S2. IC_{50} curves of the different VHs tested against M^{pro}. Dose-response curves of compounds (A) **15**, (B) **16**, (C) **12**, (D) **13**, (E) **14**, and (F) **4**. The data and IC_{50} were fitted and calculated, respectively, based on the hill equation using Rstudio.

Table S5. Inhibitory activities of virtual hits for PL^{pro}

Method

Virtual hits were tested for PL^{Pro} inhibition using a commercial assay kit from Anaspec (Fremont, USA; catalogue ID AS-72263) according to the manufacturer’s protocols. Final test concentrations of virtual hits were 20 µM with 0.2% DMSO in assay buffer. Positive control **7** was tested at a final concentration of 10 µM and 0.2% DMSO in assay buffer. Compounds and vehicle control were incubated for 15 minutes with the PL^{pro} enzyme in two well replicates before the deubiqitination substrate was added. Final concentrations of protease and substrate were 100 ng/ml and 2 µM. Two background fluorescence control wells without protease were included for each sample. After 60 minutes of incubation at room temperature, the enhanced fluorescence due to the protease-catalyzed substrate cleavage was measured at Exc/Emi 490/520 nm using a Tecan Sparks plate reader (Männedorf, Switzerland). Means of three independent experiments are presented. Statistical significance was calculated by one-way ANOVA and Dunnett’s multiple comparison test.

Table S5. Inhibitory activities of virtual hits for SARS-CoV-2 PL^{pro} measured using the commercial Anaspec assay kit including the anti SARS-CoV-2 activity and cytotoxicity of putatively active hits in two different cell lines. Significant inhibition of virtual hits was calculated with one-way ANOVA and Dunnett's multiple comparisons test (****, $p < 0.0001$; **, $p < 0.01$; *, $p < 0.05$).

Compound	SARS-CoV-2 PL^{pro} inhibition % enzyme inhibition (20 µM)	Anti SARS-CoV-2 activity		Cytotoxicity	
		Caco-2	Calu-3	Caco-2	Calu-3
		IC_{50} (µM)	IC_{50} (µM)	CC_{50} (µM)	CC_{50} (µM)
**1**, remdesivir			0.00125		>0.1
**7**, GRL-0617	96.6 ± 10.3****				
**30**, acetylshikonin	100.3 ± 9.5****	>100	>100	65.8	>100
**31**, trans-Capsaicin	32.4 ± 13.7				
**32**, lobelin	26.6 ± 11.0				
**34,** silibinin	50.3 ± 14.3*		>100		>100
**35,** honokiol	80.6 ± 9.9****	16.2	28.4	91.2	>20
**36,** magnolol	58.2 ± 11.5**	17.7	26.8	47.3	>20
**37,** oxypeucedanin hydrate	97.5 ± 14.6****		>100		>100
**38**, MolPort-001-633-809	26.1 ± 15.2				
**39**, Molport-000-428-993	26.0 ± 34.7				
**40,** Molport-001-540-175	41.5 ± 16.5				
**42,** Molport-005-910-551	82.3 ± 25.3****	7.1	>100	>100	>100
**43**, Molport-002-520-481	13.5 ± 25.5				
**44**, Molport-007-627-328	18.5 ± 16.6				
**45**, Molport-002-535-048	-2.6 ± 36.1				
**47**, Molport-039-338-319	-55.8 ± 32.4				
**48**, Molport-019-909-691	32.0 ± 4.2				

n. t. – not tested

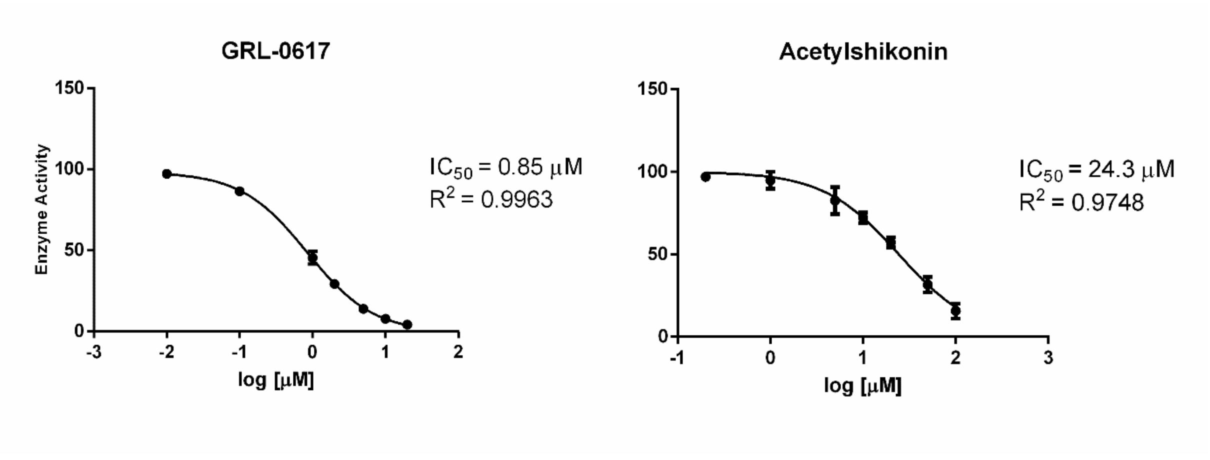

Figure S3. Concentration-response curves of positive control GRL-0617 (**7**) and acetylshikonin,(**30**) against SARS-CoV-2 PL^{pro} measured using the peptide Z-RLRGG-AMC as substrate. Fluorescence signal at 360 nm (excitation)/460 nm (emission) was immediately measured by continuous 24 points for 72 min. IC_{50}s of the compounds were determined by plotting enzyme inhibition of at least three independent experiments against seven concentrations of the test inhibitor by using the dose-response curve in GraphPad Prism.

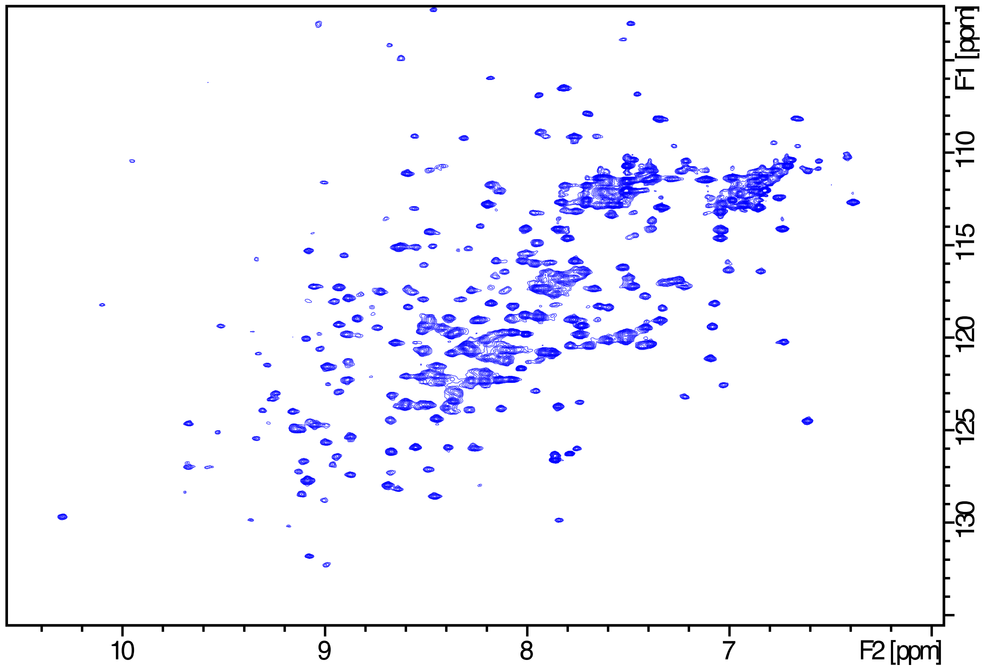

Figure S4: 2D-[^{1}H, ^{15}N]-TROSY-HSQC of 450 **μ**M of M^{pro} of SARS-CoV-2 at pH 7.6 in 50 mM phosphate buffer, 50 mM NaCl. The HSQC was acquired on a 700MHz NMR Spectrometer Bruker Advance III at 30°C.

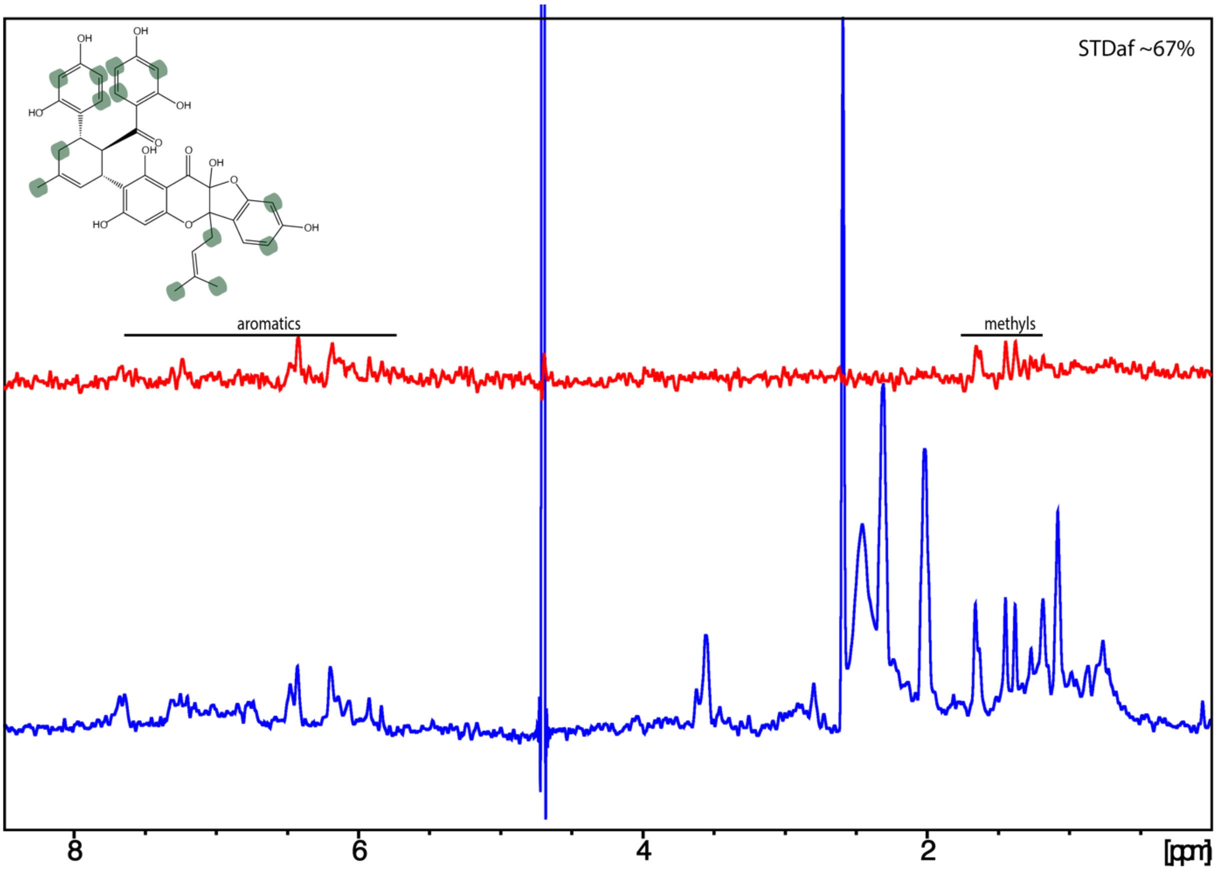

Figure S5. STD NMR of M^{pro} with compound **14.** The measurements were acquired on a 500MHz NMR Spectrometer Bruker Advance III with TCI-cryoprobe at 25°C.

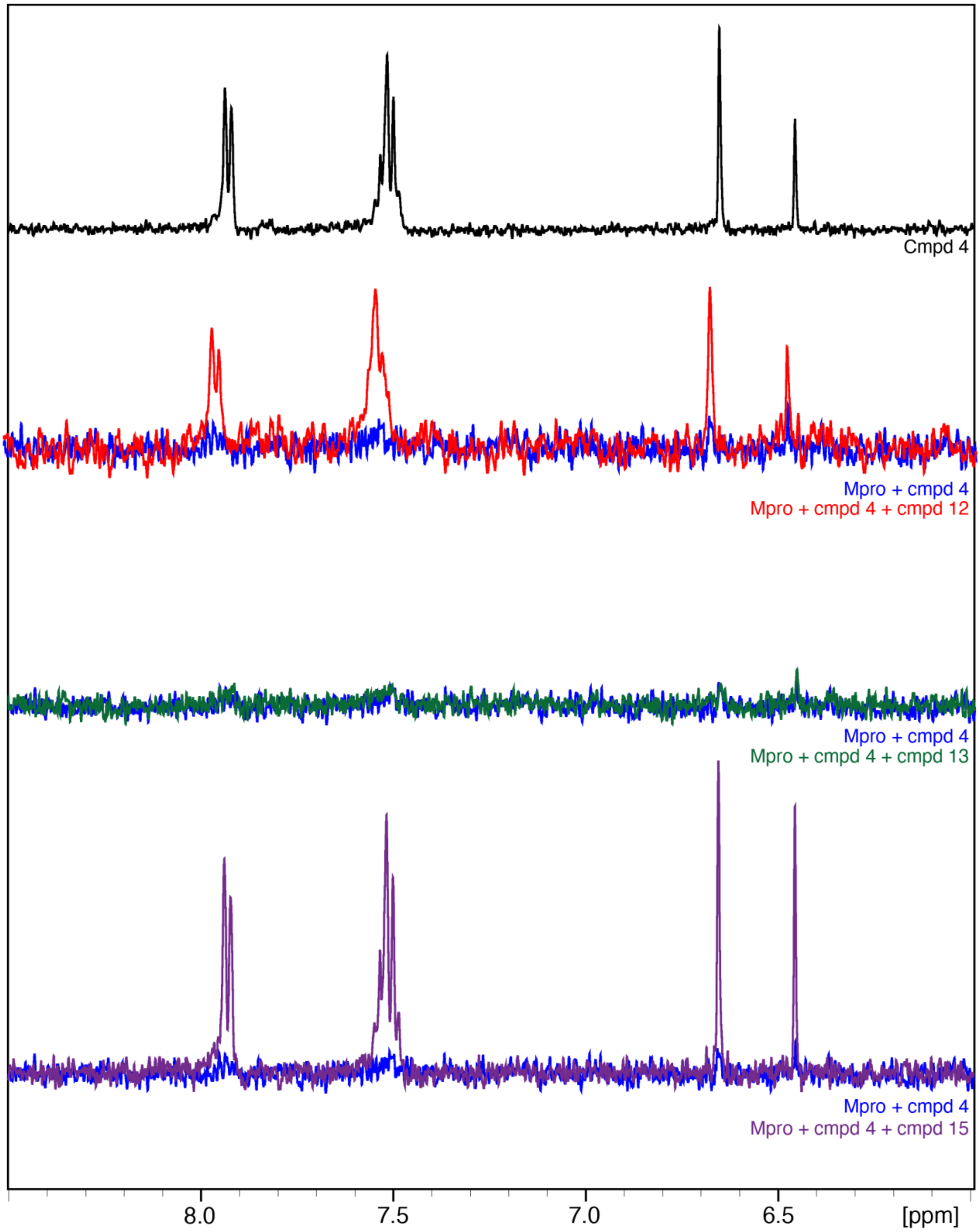

Figure S6. CPMG experiments measuread for different mixtures: compound **4**, compound **4** and M^{pro}, compound **4** with compound **12** and M^{pro}, compound **4** with compound **13** and M^{pro}, compound **4** with compound **15** and M^{pro}, depicted in black, blue, red, green and purple respectively. The sample compositions are reported in the experimental section of the main text.

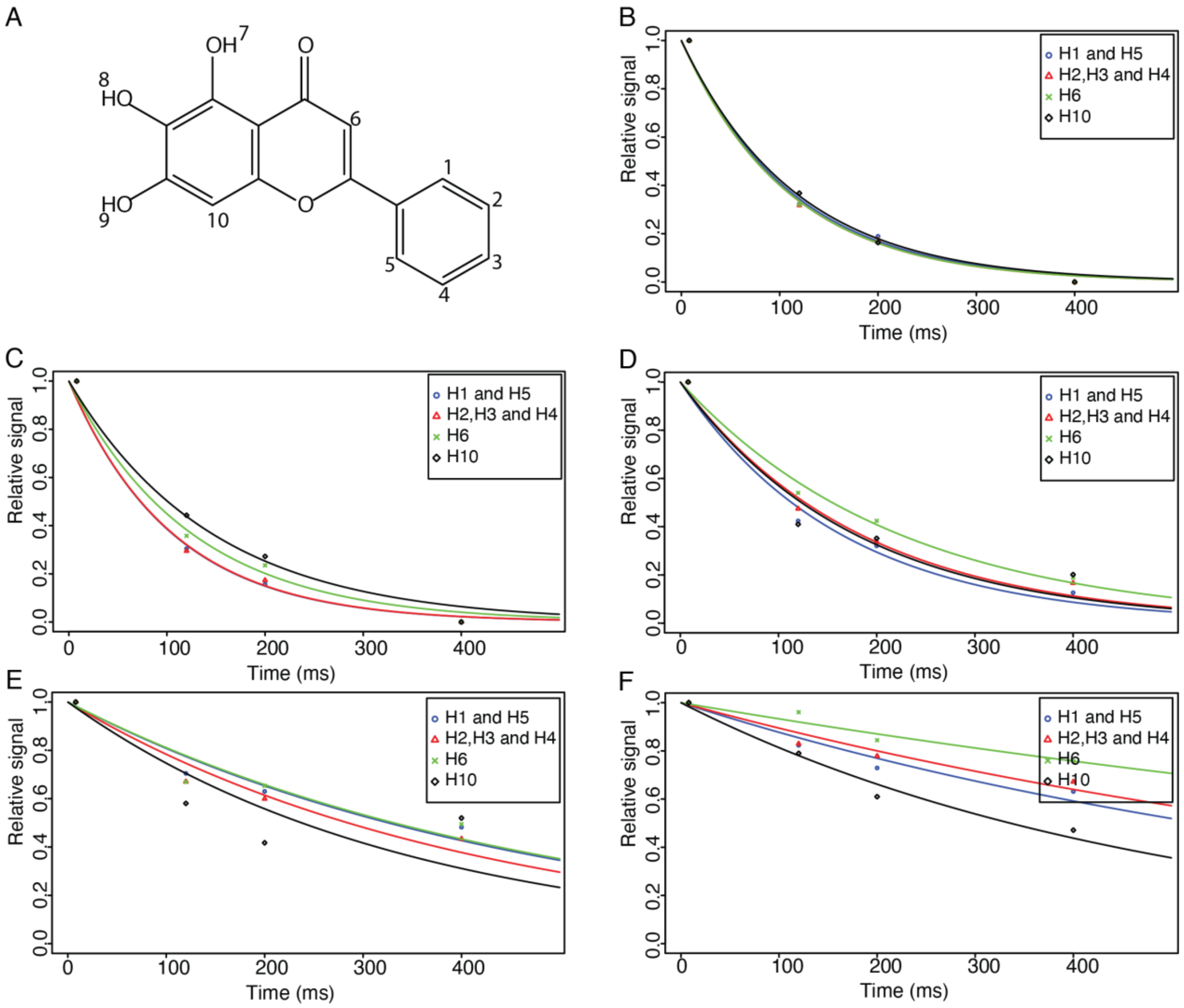

Figure S7. CPMG decay curves for each of the reporter compound **4** proton, depicted in (A), measured with the samples composed of: (B) compound **4** and M^{pro}, (C) compound **4** with compound **13** and M^{pro}, (D) compound **4** with compound **12** and M^{pro}, (E) compound **4** with compound **15** and M^{pro}, (F) compound **4**. The sample compositions are reported in the experimental section of the main text.

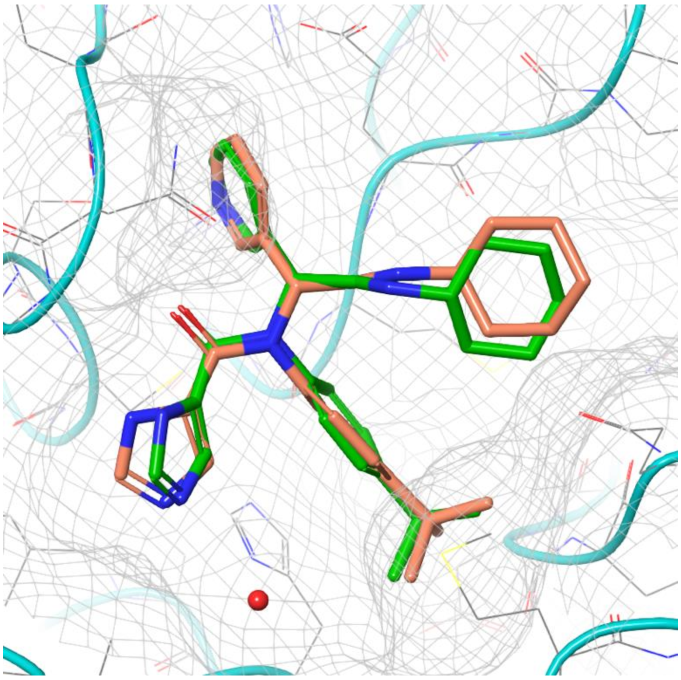

Figure S8. Comparison of the binding pose of co-crystallized ligand of 6W63 (orange) and the re-docked binding pose (green)

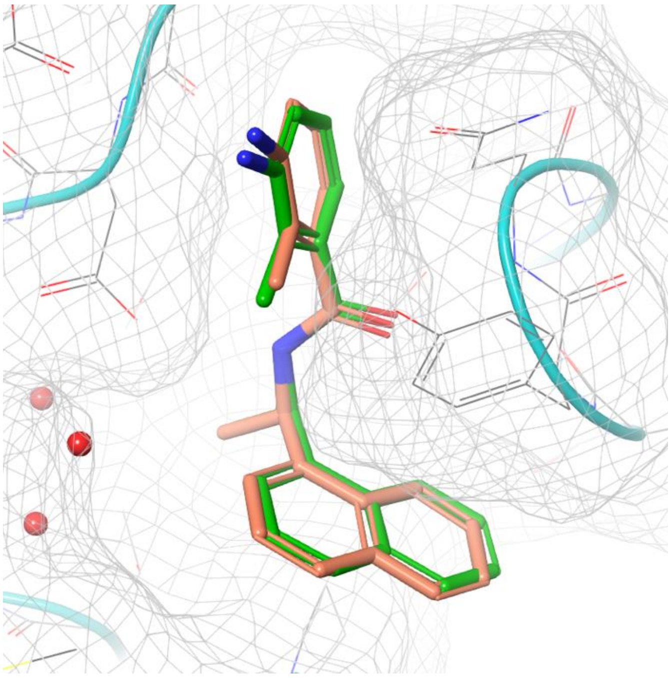

Figure S9. Comparison of the binding pose of co-crystallised ligand of 7JN2 (orange) and the re-docked binding pose (green)

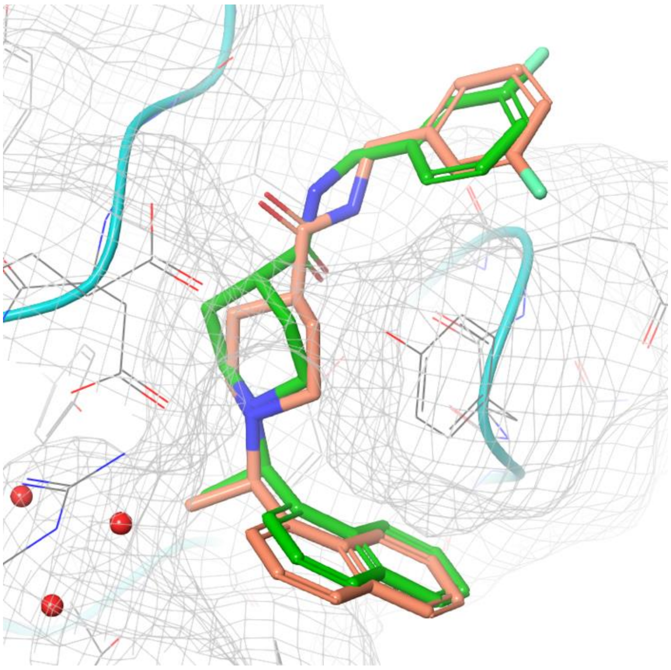

Figure S10. Comparison of the binding pose of co-crystallised ligand of 4OW0 (orange) and the re-docked binding pose (green)

Table S5. RMSD values and ChemPLP score of re-docked ligands

Protein structure (PDB)	RMSD (Å)	ChemPLP score
6W63	0,86	76,73
7JN2	0,31	91,12
4OW0	0,74	89,10

UPLC analyses of compounds **12-16**

Method

Ultra performance liquid chromatography/evaporative light scattering detector (UPLC-ELSD) was performed on Waters Acquity UPLC H-Class system equipped with a column manager, a sample manager, a quartenary solvent manager, an isocratic solvent manager, a fraction collector, a photodiode array (PDA) detector, a mass detector (QDa) and an ELSD. For analysis a Waters Acquity UPLC BEH C18 column (1.7 µm, 2.1 x 100 mm) was used. The mobile phase consits of water (A) and acetonitril (B) using gradient elution starting with 95% of B. Gradient in detail: 5% B at 0 min, from 0% to 98% B in 5 min, isocratic 98% B for 3 min. The flowrate was set at 0.3 ml/min. The software Waters Empower 3 was used for data acquistion.

UPLC-ELSD chromatogram of compound **12**

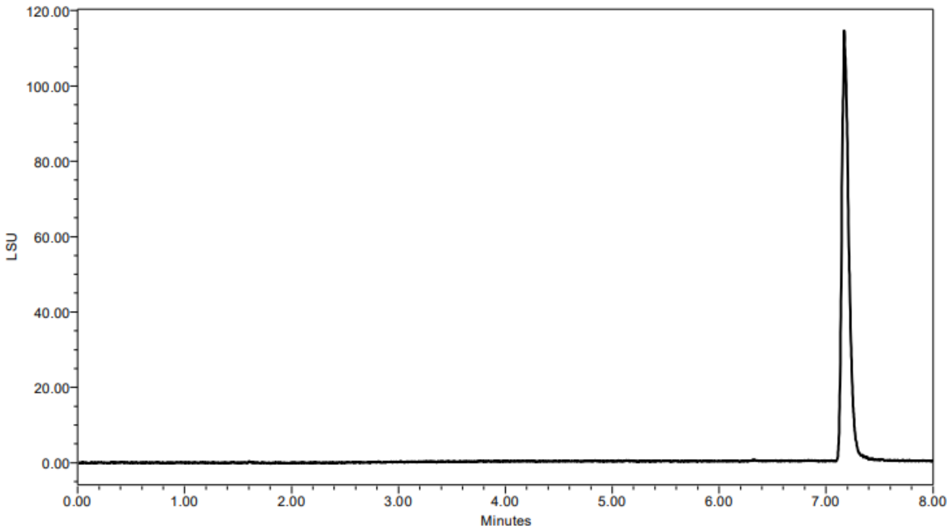

UPLC-ELSD chromatogram of compound **13**

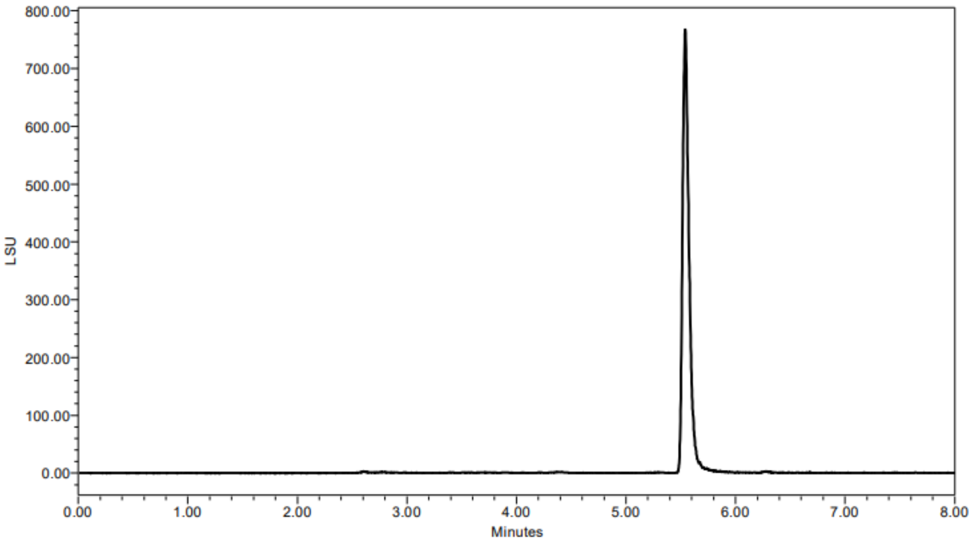

UPLC-ELSD chromatogram of compound **14**

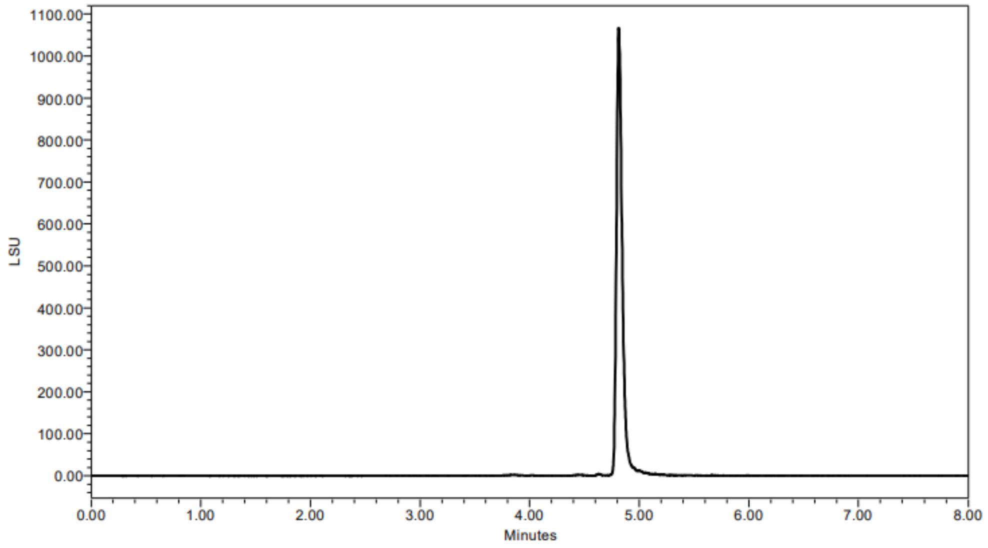

UPLC-ELSD chromatogram of compound **15**

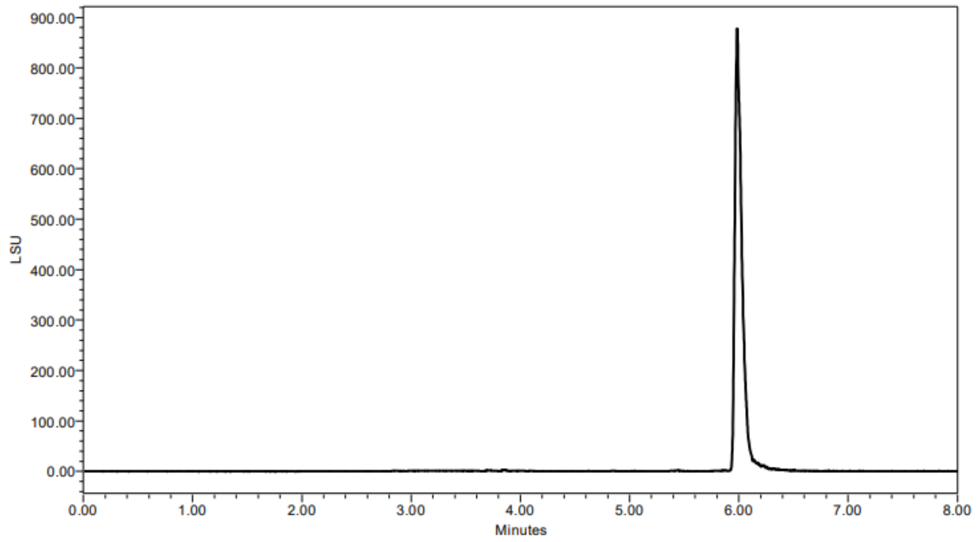

UPLC-ELSD chromatogram of compound **16**

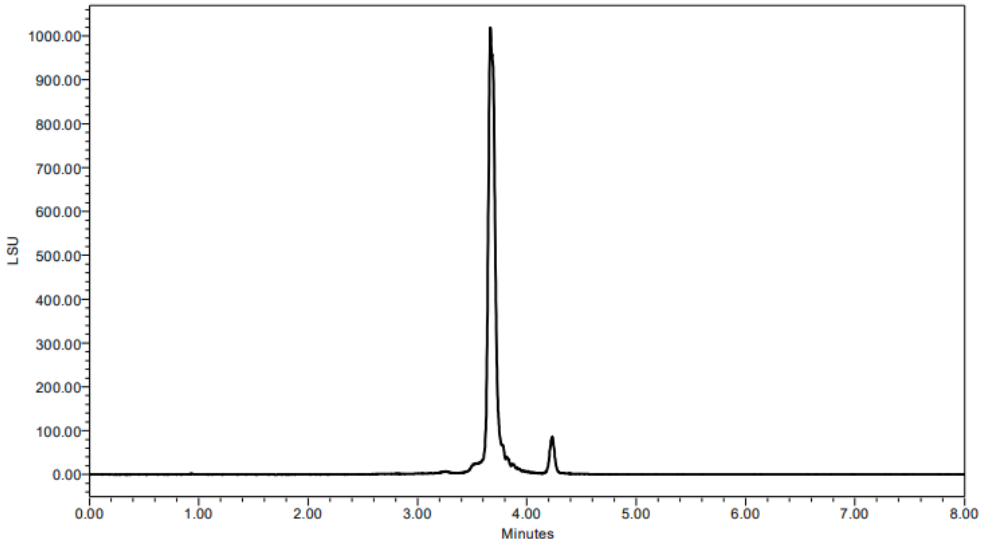

Supplement: Supplementary file 1 — np2c00843_si_001.pdf [file np2c00843_si_001.pdf]
